# Prion Aggregates Are Recruited to the Insoluble Protein Deposit (IPOD) via Myosin 2-Based Vesicular Transport

**DOI:** 10.1371/journal.pgen.1006324

**Published:** 2016-09-30

**Authors:** Rajesh Kumar, Peter P. Nawroth, Jens Tyedmers

**Affiliations:** 1 Department of Medicine I and Clinical Chemistry, Heidelberg University Hospital, Heidelberg, Germany; 2 Joint Heidelberg-IDC Translational Diabetes Program, Dept. Inner Medicine I, Heidelberg University Hospital, Germany; 3 German Center for Diabetes Research (DZD), München-Neuherberg, Germany; The University of Arizona, UNITED STATES

## Abstract

Aggregation of amyloidogenic proteins is associated with several neurodegenerative diseases. Sequestration of misfolded and aggregated proteins into specialized deposition sites may reduce their potentially detrimental properties. Yeast exhibits a distinct deposition site for amyloid aggregates termed “Insoluble PrOtein Deposit (IPOD)”, but nothing is known about the mechanism of substrate recruitment to this site. The IPOD is located directly adjacent to the Phagophore Assembly Site (PAS) where the cell initiates autophagy and the Cytoplasm-to-Vacuole Targeting (CVT) pathway destined for delivery of precursor peptidases to the vacuole. Recruitment of CVT substrates to the PAS was proposed to occur via vesicular transport on Atg9 vesicles and requires an intact actin cytoskeleton and “**SNA**P (Soluble NSF Attachment Protein) **Re**ceptor Proteins (SNARE)” protein function. It is, however, unknown how this vesicular transport machinery is linked to the actin cytoskeleton. We demonstrate that recruitment of model amyloid PrD-GFP and the CVT substrate precursor-aminopeptidase 1 (preApe1) to the IPOD or PAS, respectively, is disturbed after genetic impairment of Myo2-based actin cable transport and SNARE protein function. Rather than accumulating at the respective deposition sites, both substrates reversibly accumulated often together in the same punctate structures. Components of the CVT vesicular transport machinery including Atg8 and Atg9 as well as Myo2 partially co-localized with the joint accumulations. Thus we propose a model where vesicles, loaded with preApe1 or PrD-GFP, are recruited to tropomyosin coated actin cables via the Myo2 motor protein for delivery to the PAS and IPOD, respectively. We discuss that deposition at the IPOD is not an integrated mandatory part of the degradation pathway for amyloid aggregates, but more likely stores excess aggregates until downstream degradation pathways have the capacity to turn them over after liberation by the Hsp104 disaggregation machinery.

## Author Summary

The occurrence of large inclusions of aggregated amyloidogenic proteins is a hallmark of several neurodegenerative diseases. The most toxic species are believed to represent smaller units such as oligomers, whereas larger amyloid depositions are viewed rather cell protective. Therefore, it is important to understand how a cell recognizes and directs misfolded amyloidogenic proteins to specialized deposition sites for sequestration. We used yeast to reveal the mechanism of recruitment of a model amyloid to a specific amyloid deposition site termed IPOD. We found that during their recruitment to the IPOD, amyloid aggregates are linked to transport vesicles that are known to deliver completely unrelated substrates, namely vacuolar peptidase precursors, to a cellular site that is adjacent to the IPOD and is termed Phagophore Assembly Site (PAS) where the cell initiates autophagy. We further characterized this shared recruitment machinery and found strong evidence that it contains vesicular structures and requires a motor protein termed Myo2 that is known to transport vesicles along tropomyosin-coated actin cables. Deposition of PrD-GFP at the IPOD seems to serve more likely a temporary sequestration function when the aggregate load overwhelms downstream degradation pathways rather than an integrated step in the degradation of amyloid aggregates.

## Introduction

Protein aggregation occurs through coalescence of misfolded protein species. The cause for acquisition of an aberrant fold can be very diverse ranging from thermal, oxidative or metabolic stress, translational errors, subunit imbalance or mutations to spontaneous or induced conformational rearrangement of intrinsically unstructured proteins such as amyloids [1–3]. Albeit often indicative of protein misfolding diseases, the larger visible aggregate depositions are often cell protective rather than cytotoxic [4–6]. Thus, sequestration of aggregates into specific deposition sites was suggested to be a second line of defense to reduce the burden of freely diffusing detrimental misfolded protein species when subsequent cellular machineries that act on misfolded proteins are overwhelmed. Not surprisingly then, aggregate deposition sites exist in organisms from all kingdoms of life [7,8]. Yeast as a popular model to study processes related to protein misfolding and aggregation has at least 3 different protein quality control sites for deposition of aggregated proteins, the Juxtanuclear- or Intranuclear Quality control site (JUNQ/INQ), Q-bodies and the IPOD. While JUNQ/INQ and Q-bodies harbor more unstructured, amorphous misfolded proteins, the IPOD is regarded as a specialized deposition site for amyloid aggregates [9–12]. Amyloids are highly ordered, insoluble fibrous aggregates with a very high content of β-strands being oriented perpendicularly to the fibril axis. Their occurrence is a hallmark of several fatal neurodegenerative diseases including Parkinson’s Disease, Huntington’s Disease and various prion diseases [2]. So far, heterologously expressed amyloidogenic proteins such as the Huntington’s Disease protein fragment Htt103Q, as well as several naturally occurring yeast prions, where identified as substrates for the IPOD [10]. Studies from our and other labs using the prion-determining domain (PrD) of the yeast [*PSI*^*+*^] prion revealed that they are deposited at the IPOD in a highly ordered array of bundles of parallel, interconnected amyloid fibrils [13–15]. An additional, defining feature of the IPOD is the highly insoluble nature of the substrates, whereas substrates in JUNQ/INQ or Q-body inclusions are more soluble and have a high rate of exchange with the cytoplasm [10,12]. It was recently observed that failure of targeting of misfolded proteins to the appropriate deposition site can be associated with cellular toxicity [9,16,17]. This reveals that proper targeting of aggregates to the appropriate protein quality control compartment can be crucial for the fidelity of cells. Thus, the cell must be able to differentiate between different types of aggregates to direct them to the spatially separated deposition sites.

Following this concept, initial studies have revealed components that are essential for substrate targeting to either the JUNQ/INQ or Q-body sites. Those include the small heat shock protein Hsp42, the related protein Btn2, Sis1 as well as the Hsp70-Hsp90 chaperone network [9,18–20]. For the IPOD, which is located at the vacuolar membrane adjacent to the Phagophore Assembly Site (PAS) [15] where the cell initiates biogenesis of autophagosomes and CVT vesicles [21,22], not much is known about substrate recognition and targeting mechanisms. Nevertheless, it is known that several molecular chaperones can interact with amyloid aggregates at the IPOD [14,23,24] or in amyloid aggregates more dispersed throughout the cytoplasm. For the latter case, next to molecular chaperones, additional proteins including components of the actin cytoskeleton and/or endocytosis machinery, have been found associated with amyloid aggregates [25–30]. However, it is hard to predict only from their presence in aggregates whether some of these amyloid-binding proteins are also involved in substrate recruitment to the IPOD.

Therefore, we employed an unbiased proteomics approach and identified, amongst others, proteins of the actin cable-based transport machinery and the machinery for vesicle fusions to bind to amyloid fibrils of the model substrate PrD-GFP and to be required for their faithful recruitment to the IPOD *in vivo*. Impairment of this recruitment machinery led not only to the accumulation of PrD-GFP aggregates, but also to co-accumulation of vacuolar precursor peptidases destined for the IPOD-adjacent PAS, suggesting that both are recruited through a very similar machinery.

## Results

### Identification of binding partners of PrD amyloid fibrils

**Fig 1:**
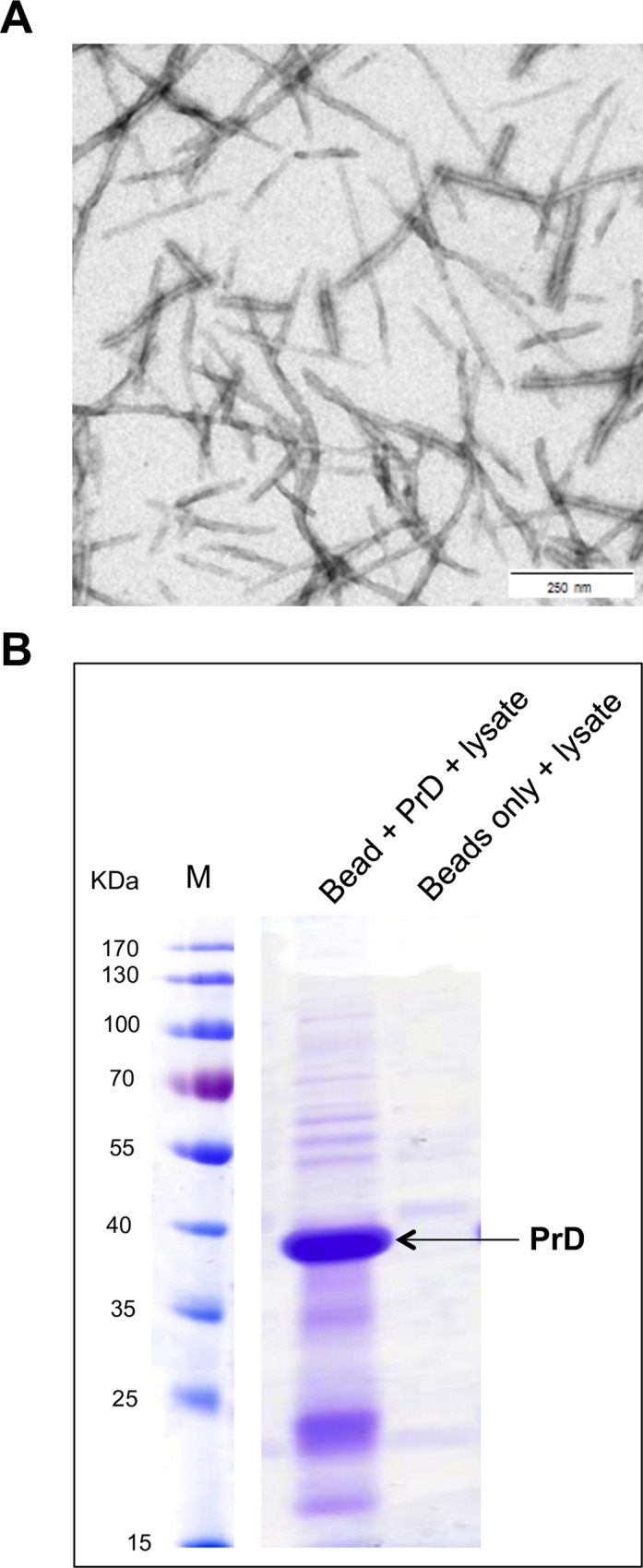
Identification of PrD amyloid binding proteins. (**A**) Electron Microscopy (EM) of PrD amyloid fibers prepared from biotin-labeled and unlabeled recombinant PrD at a ratio of 1:50 after negative staining with uranyl acetate at 50000X magnification. Scale bar, 250 nm. (**B**) Biotin-labeled PrD fibers were immobilized to avidin-coated magnetic beads and incubated with [*PSI*^+^] cell lysates. Proteins bound to the resin were eluted by boiling in SDS sample buffer, subjected to SDS-PAGE and stained with Coomassie. The left lane shows the eluate from PrD-fiber containing resin, the right lane an eluate from a control resin without PrD fibers. Eluted proteins were subsequently identified by mass spectrometry (S1 Table).

Amyloid aggregates have previously been observed in yeast to accumulate in a particular deposition site termed IPOD [10]. However, little is known about mechanisms of their recognition and recruitment to the IPOD. Therefore, we used *in vitro* assembled recombinant PrD amyloid fibrils immobilized through a biotin moiety to identify amyloid binding factors from yeast cell lysates. PrD corresponds to the well-characterized prion domain of Sup35 responsible for the [*PSI*^*+*^] prion [31–33]. We immobilized PrD fibers (Fig 1A) to magnetic avidin beads and incubated them with [*PSI*^*+*^] yeast cell lysates. As a control for unspecific binding, avidin-coated beads without any immobilized PrD were used. Proteins bound to the resin were eluted and subjected to SDS PAGE (Fig 1B) prior to identification by mass spectrometry (S1 Table). Several of the proteins enriched in the resin with the PrD bait, highlighted in green in S1 Table, had previously been described to interact with Sup35 or its PrD [25,34–36], validating our method. Although we did not find all known interactors of [*PSI*^+^] aggregates, which indicates that our method is not quantitative, we found several proteins that were not yet known to interact with [*PSI*^+^] aggregates, including tropomyosin1/2 and SEC genes.

The major isoform of tropomyosin, represented by the two homologs Tpm1 and Tpm2 in yeast, binds to actin filaments, thereby influencing transport processes along actin cables [37]. Therefore we wondered whether Tpm1/2 is involved in the recruitment of PrD-GFP to the IPOD. First, we tested by co-localization studies whether Tpm1/2-mCherry was detectable in the IPOD deposition site itself, which was not the case (S1 Fig). Thus if the interaction of Tpm1/2 with PrD fibers was specific, it may *in vivo* not take place at the IPOD, but more transiently, for example in a step during recruitment of PrD to the IPOD.

### Tropomyosin and Myo2 functions are required for proper accumulation of PrD-GFP at the IPOD

**Fig 2:**
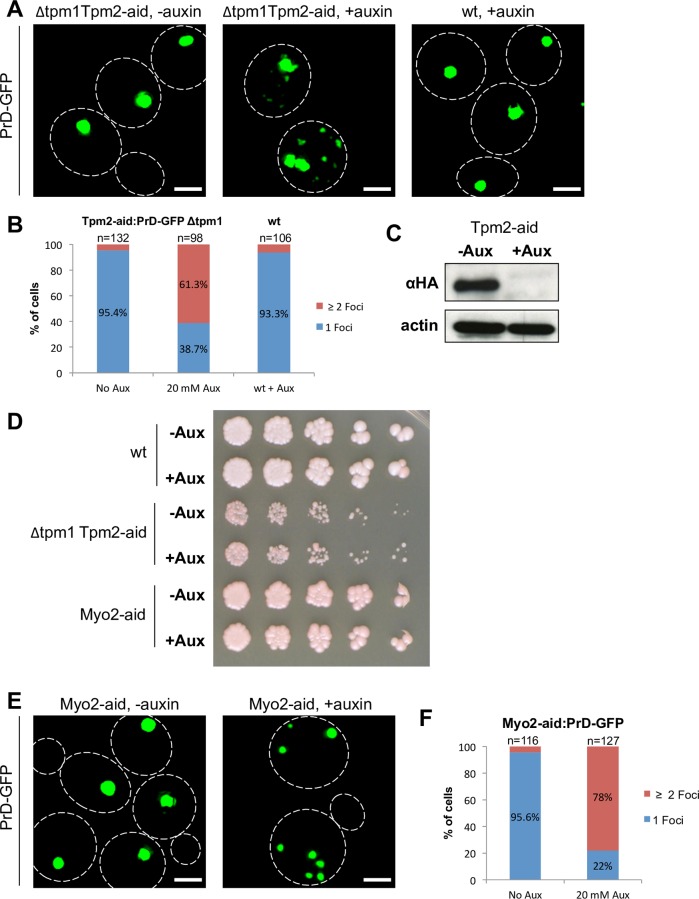
Tropomyosin and Myo2 are essential for proper accumulation of PrD-GFP at the IPOD. (**A**) PrD-GFP was induced with galactose for 6 hours in a [*PSI*^+^] strain containing a deletion of Tpm1 and a C-terminal aid tag in Tpm2 (RK1c) (left and middle panel) or being wild type (wt) (RK1), in the absence or presence of 20 mM of auxin as indicated. Cells were fixed and analyzed by fluorescence microscopy. (**B, F**) Quantification of PrD-GFP foci upon depletion of Tpm1/2 (B) or Myo2 (F). Frequencies of cells with 1 single focus or more than 1 foci are given in %. (**C**) Western blot of the *tpm1Δ* Tpm2-aid strain after treatment as in (A) with an antibody against an HA-tag present in the aid-tag. An anti-actin antibody served as loading control. (**D**) Wild type (wt), *tpm1Δ* Tpm2-aid and Myo2-aid strains were treated as in (A) prior to serial dilution and a spotting test for cell viability. Cells were grown on YPD plates for 2–3 days. (**E)** Same experiment as in (A), but with a [*PSI*^+^] strain with a C-terminal aid-tag in MYO2 (E) (RK1e). Scale bar = 2 μm.

If tropomyosin is required for the recruitment of PrD-GFP to the IPOD, a lack of the protein should impair proper deposition. To test this, we depleted tropomyosin function from a [*PSI*^+^] strain that carried PrD-GFP under control of a galactose inducible promoter. As a double deletion of Tpm1 and Tpm2 would be lethal, we conditionally reduced the levels of Tpm2 in a tpm1 deletion strain using an “Auxin Inducible Degron (aid)” tag strategy [38]. This tag allows for targeting of the protein that carries it to proteasomal degradation upon addition of the plant hormone auxin. We induced PrD-GFP expression in galactose-based media for 6 hours in the constant presence of auxin. Although the depletion of tpm2-aid would not require 6 hours, we incubated the cells with auxin for the entire time to ensure that the tpm2 levels were always low during PrD-GFP synthesis. As a control, we grew the same strain under identical conditions, but in the absence of auxin. Then, we withdrew aliquots, fixed the cells and subjected them to fluorescence microscopy to monitor the aggregation patterns of PrD-GFP. In the control, 95% of the cells had one single large PrD-GFP aggregate (Fig 2A, left panel and Fig 2B) as previously described [15]. In contrast, the depletion of tpm1/2 led to multiple PrD-GFP foci in 61% of the cells (Fig 2A, middle panel, and Fig 2B). To exclude that this phenotype was a side effect of auxin itself, we also incubated cells lacking an aid-tag with auxin and found only 1 single PrD-GFP aggregate per cell, as expected (Fig 2A, right panel and Fig 2B). Analysis of the protein levels of Tpm2-aid after 6 hours of auxin by Western Blotting (Fig 2C) confirmed a strong reduction. Furthermore, a spotting test of serial dilutions of the same cultures after 6 hours of galactose induction in the presence of auxin revealed that the viability of the cells was not affected (Fig 2D). In contrast, when the cells were grown for two days in the presence of auxin on SD plates (S2A Fig), we observed strongly reduced growth, further confirming that depletion of the essential Tpm1/2 function is very efficient. In summary, depletion of the tropomyosin function interfered with the proper occurrence of one single IPOD harboring PrD-GFP.

Tropomyosin marks actin cables to allow for more efficient interactions with myosin typeV proteins such as Myo2 [39]. In conjunction with tropomyosin, Myo2 is also involved in asymmetric inheritance of different types of aggregates including the Huntington’s disease protein Htt103Q in yeast [29,40]. Due to this functional connection, we also included the essential protein Myo2 into our analysis. Just like Tpm2, we fused the protein with an aid-tag, added auxin and confirmed its reduction by Western Blotting (S2B Fig). Depletion for 6 hours during PrD-GFP induction with galactose did not interfere with cell viability (Fig 2D, bottom panel), however prolonged incubation with auxin did (S2A Fig). Viability of cells after 6 hours of depletion of Myo2 and Tpm1/2 was also confirmed using the more quantitative colony forming units assay (S2C and S2D Fig). Fluorescence microscopy revealed that depletion of Myo2 had a very similar phenotype as compared to Tpm1/2 (Fig 2E and 2F), suggesting that Myo2 function is also crucial for proper accumulation of PrD-GFP at the IPOD.

### Restoration of PrD-GFP localization upon ceasing depletion of Myo2

**Fig 3:**
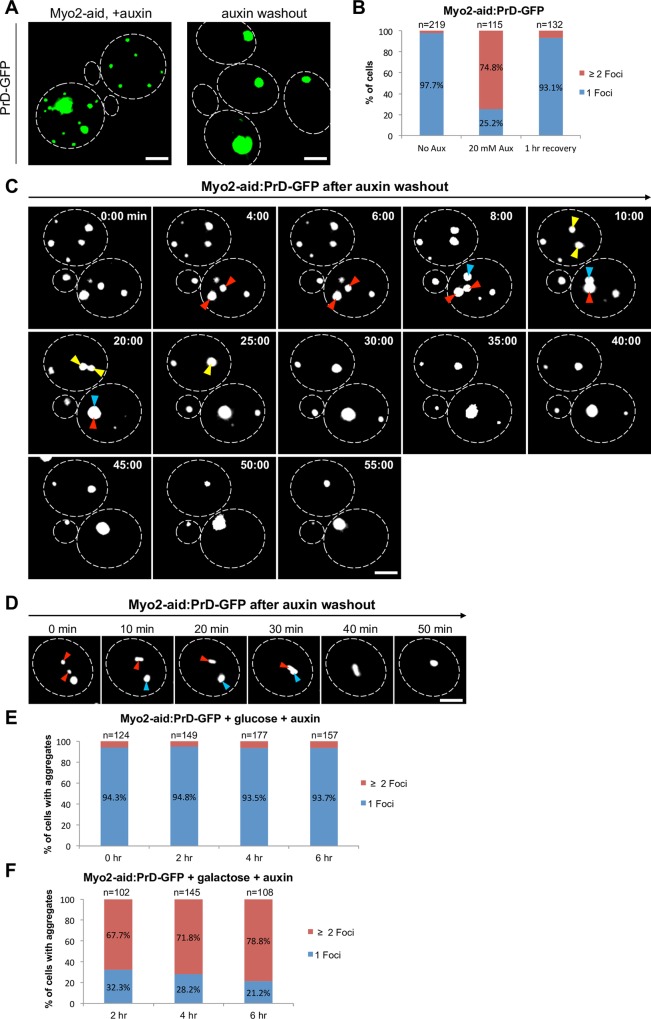
Washout of auxin to rescue Myo2 function restores IPOD localization of PrD-GFP. (**A**) PrD-GFP was induced with galactose for 6 hours in the presence of 20 mM auxin in a [*PSI*^+^] strain with a C-terminal aid-tag in MYO2 (RK1e) (left panel). Subsequently, cells were pelleted, resuspended in YPD without auxin to rescue Myo2 function and further incubated for 60 min in the presence of glucose prior to fixation and fluorescence microscopy. (**B**) Quantification of PrD-GFP localization in either 1 or more than 1 foci in % in the absence or presence of 20 mM auxin or after auxin washout (1 hr recovery). (**C**) PrD-GFP was induced with galactose for 6 hours in the presence of 20 mM auxin in a [*PSI*^+^] Myo2-aid strain. Subsequently, cells were pelleted and placed onto an agarose pad containing glucose instead of galactose on a microscope slide for time-lapse microscopy. A z-stack with a step width of 0.3 μm was taken every 2–5 min (**D**) A single cell from an experiment as in C to highlight fusion events of PrD-GFP foci. **(E)** PrD-GFP was induced with galactose for 6 hours in the absence of auxin in a [*PSI*^+^] Myo2-aid strain to allow for IPOD formation. Subsequently, cells were pelleted, resuspended in YPD in the presence of 20 mM of auxin and further incubated for up to 6 hours. At indicated time points, aliquots were withdrawn, fixed and analyzed by fluorescence microscopy. PrD-GFP localization in either 1 or more than 1 foci was determined from cells that still carried PrD-GFP aggregates and plotted as %. **(F)** PrD-GFP was induced with galactose in the presence of 20 mM auxin in a [*PSI*^+^] Myo2-aid strain (RK1e). After the indicated times, aliquots were withdrawn, fixed and analyzed by fluorescence microscopy. PrD-GFP localization in either 1 or more than 1 foci was determined and plotted as %. Scale bar = 2 μm.

Loss of tropomyosin and Myo2 function led to multiple PrD-GFP aggregates dispersed throughout the cytpoplasm instead of 1 single IPOD deposition. If these genes really promoted aggregate coalescence and recruitment to the central IPOD, ceasing the auxin-induced depletion should readily rescue the observed defect. Since the two proteins are functionally linked and caused the same phenotype, we focused on Myo2 depletion in the following experiments. We washed out auxin after 6 hours of Myo2-depletion and further incubated the cells in the absence of both auxin and galactose for up to 1 hour to prevent ongoing protein synthesis and consequently to be able to follow only the fate of pre-existing PrD-GFP aggregates. Whereas the IPOD was initially disrupted in 75% of the cells, 93% of the cells had one single PrD-GFP deposition again after 1 hour of auxin wash-out (Fig 3A and 3B). To further confirm that we observed refusion of the dispersed PrD-GFP foci rather than their degradation, we did time-lapse microscopy immediately after the wash out of auxin in the absence of galactose in glucose based media (see methods) and followed individual cells for 60 min by taking a z-stack every 2–5 minutes and merge the layers into one image. This strategy of image display does not allow for a nice differentiation between cytoplasmic, vacuolar and nuclear fluorescence, but it was necessary to monitor all aggregates present in the cell. Next to a strong reduction in the number of PrD-GFP foci to mostly 1 single aggregate (Fig 3C), we could also directly observe refusion of PrD-GFP aggregates (Fig 3C and 3D). S3A Fig shows a video of such a time-lapse experiment. A similar refusion in 86% of the cells was also observed Tpm1/2 depletion was ceased in a similar way as for Myo2 (S3B Fig).

Although Myo2 and Tpm1/2 are known to mediate different cellular transport processes [41], we could not yet rule out that with regard to the IPOD, their function might be to maintain the integrity of the IPOD rather than to recruit PrD-GFP aggregates to this site. To differentiate between these two possibilities, we tested the effect of Myo2 depletion after the PrD-GFP IPOD had already formed. We induced PrD-GFP for 6 hours with galactose in the absence of auxin, shifted the cells to a glucose-based media to abolish further PrD-GFP expression and added auxin. After 0, 2, 4 and 6 hours, we withdrew aliquots for fluorescence microscopy (S4A Fig) and quantified the number of cells with one single PrD-GFP aggregate versus multiple ones (Fig 3E). We did not observe any disruption of pre-existing IPOD accumulations, supporting the hypothesis that Myo2 is involved in recruitment of PrD-GFP aggregates to the IPOD. We note that the preexisting PrD-GFP aggregates slowly decayed over time (S4A Fig), which we further explored in the experiments described in the last section of the results.

To further differentiate between recruitment function versus maintenance of IPOD integrity, we tested whether depletion of Myo2 leads immediately to the formation of multiple PrD-GFP aggregates or a transient appearance of one central IPOD aggregate before multiple PrD-GFP foci would emerge. We induced PrD-GFP expression with galactose in the presence of auxin, withdrew aliquots after 2, 4 and 6 hours (S4B Fig) and quantified the number of PrD-GFP foci per cell (Fig 3F). We observed for all time points that the vast majority of cells had two or more PrD-GFP foci (68%–78%) (Fig 3F). Together, these results favor the hypothesis that disruption of Myo2 function leads to a defect in the recruitment of PrD-GFP to the IPOD rather than interfering with its integrity.

Next we asked whether Myo2 is also involved in recruitment of other amyloid substrates to the IPOD and tested the additional bona fide IPOD substrates Ure2-YFP, Htt103Q-CFP and Rnq1-GFP [10]. We used identical conditions as for the PrD-GFP substrate in strains that express the corresponding other substrates instead of PrD-GFP and found the same phenotype of accumulation of multiple fluorescent foci in the majority of the cells after depletion of Myo2. Upon ceasing depletion, the multiple foci re-fused to 1 single IPOD inclusion in most of the cells (S5A–S5L Fig). In analogy to the PrD-GFP substrate (compare Fig 3E and S4A Fig), we also tested for one additional substrate, namely Rnq1-GFP, if an IPOD pre-formed by this substrate in the absence of auxin would stay intact over a period of 6 hours when the cells were depleted from Myo2 after the IPOD had formed (S6A Fig). As for PrD-GFP, we did not observe any disruption of pre-existing IPOD accumulations (S6B Fig).

Next, we monitored the simultaneous recruitment of two different amyloid substrates in the same strain and asked if both substrates would get recruited to the same site after restoration of Myo2 function. We used a Myo2-aid strain that expressed Ure2-YFP and Htt103Q-CFP, induced both substrates with galactose for 6 hours in the presence of auxin, washed out auxin and shifted the cells to glucose based media. After Myo2 depletion, we observed again multiple foci of both substrates as expected that co-localized with each other (S6C Fig, middle panel). After restoring Myo2 function, the multiple foci consisting of the two different substrates accumulated again in one single IPOD (S6C Fig, lower panel). This further demonstrates that there is one unique IPOD site to which different substrates are recruited in a Myo2 dependent manner.

Together, these data described above strongly support the interpretation that depletion of Myo2 and tropomyosin function impaired the recruitment of amyloid aggregate substrates to the IPOD site rather than interfering with the integrity of it.

### Depletion of Myo2 function results in accumulation of Atg8 and the CVT substrate preApeI in multiple cluster

**Fig 4:**
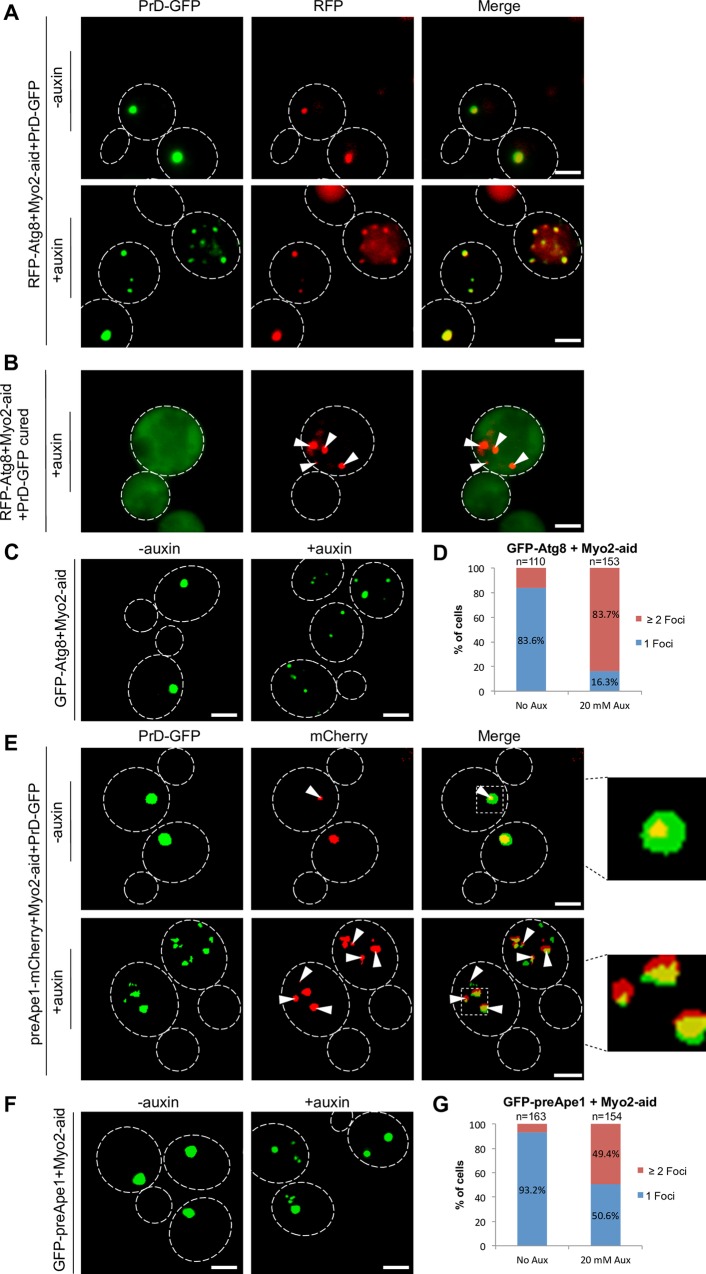
Depletion of Myo2 results in co-accumulation of Atg8 and preApe1 together with PrD-GFP foci. (**A**) PrD-GFP was induced with galactose for 6 hours in the absence or presence of 20 mM auxin in a [*PSI*^+^] Myo2-aid strain that carried a plasmid coding for RFP-Atg8 (RK1e+pRFP-Atg8). Cells were fixed and analyzed by fluorescence microscopy in the GFP and mCherry channels. Co-localization:–auxin: 79%, n = 114 foci; +auxin: 89%, n = 96 foci. **(B)** Same experiment as in (A) in the same strain after curing of the [*PSI*^+^] prion with GdnHCl. (**C**) Same experiment as in (A), but in a [*PSI*^+^] Myo2-aid strain that did not express PrD-GFP, but GFP-Atg8 as a genomic fusion under control of the Gal1 promoter (RK5f). **(D)** Quantification of GFP-Atg8 foci upon depletion of Myo2. Frequencies of cells with 1 single focus or more than 1 foci are given in %. (**E**) Same experiment as in (A), but in a [*PSI*^+^] Myo2-aid strain with a genomic C-terminal mCherry fusion to Ape1 (RK1e-APE1-mCh) instead of RFP-Atg8. Co-localization:–auxin: 60%, n = 98 foci; +auxin: 65%, n = 234 foci. (**F**) Same experiment as in (A), but in a [*PSI*^+^] Myo2-aid strain that did not express PrD-GFP, but a genomic N-terminal GFP fusion of Ape1 controlled by a Gal promoter (RK5e). **(G)** Quantification of GFP-preApe1 foci upon depletion of Myo2. Frequencies of cells with 1 single focus or more than 1 foci are given in %. Scale bar = 2 μm.

Depletion of Myo2 led to a reversible accumulation of multiple PrD-GFP aggregates destined for deposition at the IPOD. The IPOD is located in a perivacuolar site adjacent to the PAS [10,15] where the cell initiates formation of autophagosomes and CVT-vesicles. The CVT-pathway mediates translocation of vacuolar peptidase precursors into the lumen of the vacuole via an autophagy related mechanism. Prior to the formation of CVT vesicles, several structural components including the PAS marker Atg8 as well as the peptidase precursors accumulate at the PAS [22]. Interestingly, disturbing the integrity of the actin cytoskeleton caused a failure of corresponding cells to recruit Atg8 to the PAS [42]. This led to the hypothesis that actin cable based transport is involved in recruiting proteins to the PAS, but it remained unclear how the substrates are linked to the actin cytoskeleton [43]. Since we found that Myo2 and Tpm1/2, both involved in actin cable transport, are required for the recruitment of PrD-GFP to the IPOD directly adjacent to the PAS, we asked whether recruitment of RFP-Atg8 to the PAS is still functional in cells with reduced Myo2 function. We repeated the depletion of Myo2 as described, but in a strain that contained in addition to PrD-GFP a plasmid coding for RFP-Atg8. In the absence of auxin, RFP-Atg8 formed a single focus that co-localized with PrD-GFP at the IPOD in 79% of the cells (Fig 4A, upper panel) as expected [15]. After Myo2 depletion however, multiple foci of RFP-Atg8 and PrD-GFP were observed (Fig 4A, lower panel). Intriguingly, 89% of the PrD-GFP foci co-localized with RFP-Atg8. Amyloid aggregates tend to be sticky and can potentially co-capture other proteins [44]. This left the possibility that RFP-Atg8 formed multiple foci because the protein was co-captured by PrD-GFP aggregates. To rule this out, we cured the strain that revealed co-localization of PrD-GFP with RFP-Atg8 with 5 mM GdHCl prior to Myo2 depletion. This curing is known to eliminate prions and leads to diffusely distributed PrD-GFP in the cytoplasm [15,45]. When we monitored the cured cells after depletion of Myo2, we found PrD-GFP distributed diffusely in the cytoplasm as expected, whereas RFP-Atg8 was still forming multiple foci in the presence of auxin (Fig 4B). Thus PrD-GFP and RFP-Atg8 do not just co-aggregate. PrD-GFP foci refused to 1 single focal aggregate again after ceasing Myo2 depletion. Therefore, we wondered whether this was also true for RFP-Atg8. Unfortunately, we were not able to study a possible refusion of RFP-Atg8 foci together with the co-localizing PrD-GFP foci in the same strain because RFP/mCherry bleached very fast, especially when expressed at low levels like here. Therefore, we used a corresponding genomic N-terminal GFP fusion to Atg8 under control of the Gal1 promoter. Again, depletion of Myo2 led to multiple GFP-Atg8 foci (Fig 4C and 4D), which re-fused to one focal aggregate in 79% of the cells after ceasing Myo2 depletion (S7A–S7C Fig).

After we observed that RFP-Atg8 accumulated in the same locations as PrD-GFP aggregates upon depletion of Myo2 function, we wondered how a substrate for the CVT pathway would behave. The vacuolar peptidase precursor preApe1 is such a substrate that accumulates in multimeric complexes at the PAS prior to CVT vesicle formation and delivery to the vacuolar lumen. Interestingly, also preApeI recruitment to the PAS was previously found to require an intact actin cytoskeleton [43].

We used again the galactose-inducible PrD-GFP strain that allowed for conditional depletion of Myo2, transformed it with a preApe1-mCherry fusion and performed the experiment as it was done with RFP-Atg8. In the control without auxin, we observed one single red focus of preApeI-mCherry co-localizing with PrD-GFP in 60% of the cells (Fig 4E). We note that against our expectations [10,15,46], the single PrD-GFP focus was not co-localizing with the single preApe1 focus in 40% of the cells for unknown reasons in control cells. Possibly, the corresponding mCherry tag in preApe1-mCherry is slightly interfering with the properties of the protein. Auxin induced depletion of Myo2 led to multiple foci of both PrD-GFP and preApeI-mCherry. 65% of the PrD-GFP aggregates were associated with preApe1-mCherry (Fig 4E). We also note that the fluorescent foci did not superimpose as well as compared to RFP-Atg8. To demonstrate that preApe1 would also mislocalize after Myo2 depletion in the absence of the potentially sticky PrD-GFP aggregates, we repeated the experiment with an N-terminal genomic GFP fusion to preApe1 under control of the Gal promoter in the absence of PrD-GFP. In the control without auxin, a normal localization of preApe1 to 1 single fluorescent focus representing the PAS was observed. However, after depletion of Myo2, multiple GFP-preApe1 foci emerged (Fig 4F) in 49% of the cells (Fig 4G), confirming that Myo2 is required for faithful targeting of preApe1 to the PAS.

### Characterization of the joint accumulations of PrD-GFP, Atg8 and preApe1

**Fig 5:**
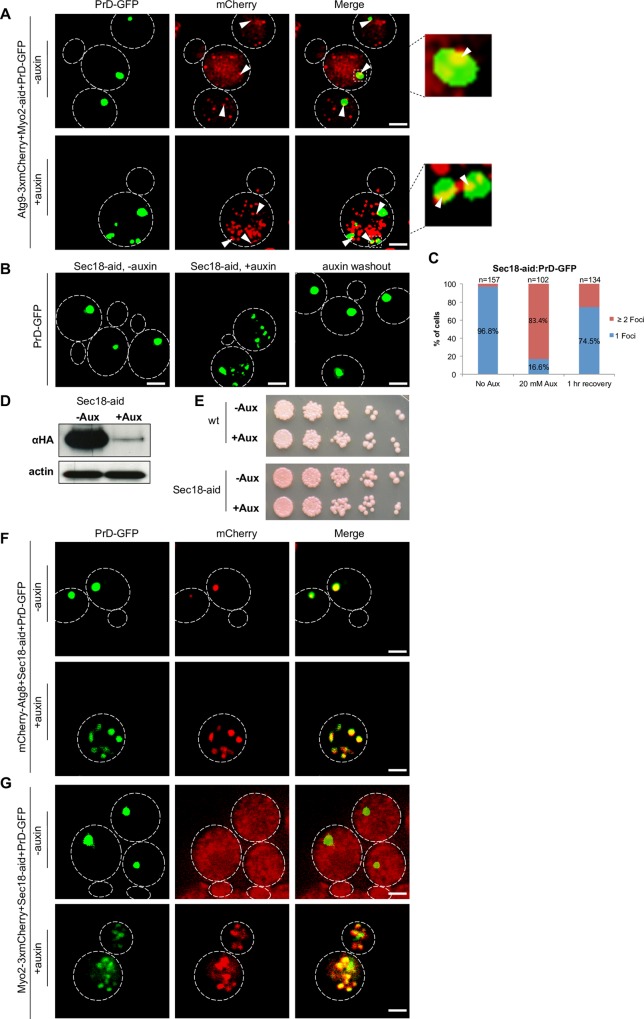
Characterization of the joint accumulations of PrD-GFP, Atg8 and preApe1. (**A**) PrD-GFP was induced with galactose for 6 hours in the absence or presence of 20 mM auxin in a [*PSI*^+^] Myo2-aid strain with a C-terminal 3xmCherry-tag in the endogenous ATG9 (RK1e-ATG9-3xmCh). Cells were fixed with and analyzed by fluorescence microscopy in the GFP and mCherry channels. Co-localization:–auxin: 78%, n = 116 foci; +auxin: 77%, n = 205 foci. (**B**) PrD-GFP was induced with galactose for 6 hours in the absence or presence of 20 mM auxin in a [*PSI*^+^] strain that carried a C-terminal aid-tag in SEC18 (RK1g) (left and middle panel). Subsequently, cells were pelleted, resuspended in YPD without auxin to restore Sec18 function and incubated further for 60 min (auxin washout, right panel) prior to fixation and fluorescence microscopy. (**C**) Quantification of PrD-GFP localization in either 1 or more than 1 foci in % in the absence or presence of 20 mM auxin or after auxin washout (1 hr recovery). (**D**) Western blot analysis of a [*PSI*^+^] Sec18-aid strain without (-Aux) or after depletion (+Aux) of Sec18 with an antibody against an HA-tag present in the aid-tag. An anti-actin antibody served as loading control. (**E**) A [*PSI*^+^] wild type (wt) and a Sec18-aid strain were treated as described in (A) before serial dilution and spotting onto YPD plates and growth for 2 days to test for viability. (**F**) PrD-GFP was induced with galactose for 6 hours in the absence or presence of 20 mM auxin in a [*PSI*^+^] Sec18-aid strain with an N-terminal mCherry-Atg8 fusion under control of the Atg8 promoter (RK1g+pmCh-ATG8) prior to fixation and fluorescence microscopy in the GFP and mCherry channels. Co-localization:–auxin: 76%, n = 105 foci; +auxin: 79%, n = 108 foci. **(G)** The experiment was done as in (F), but in a [*PSI*^+^] Sec18-aid strain with a C-terminal 3xmCherry-tag in the endogenous MYO2 (RK1g-MYO2-3xmCh). Co-localization: +auxin: 74%, n = 238 foci. Scale bar = 2 μm.

A role of actin in recruitment of preApe1 to the PAS in non-starved, growing cells has been observed previously. From those studies, it was proposed that the so called CVT complex consisting of preApe1 together with its specific receptor is recruited to Atg9 vesicles, which are then targeted to the PAS with the aid of actin cables. Consistently, disruption of the actin cytoskeleton caused failure of preApe1, but also Atg9, to be recruited to the PAS. However, the linking factor between these Atg9 vesicles and actin that would enable cargo movement remained unknown [43]. Upon Myo2 depletion, we observed preApe1 to accumulate in multiple punctate structures that often partially overlapped with the prion aggregates, which also co-localized with RFP-Atg8. This suggested that all three proteins localize to similar structures. To further reveal the nature of these structures, we asked whether Atg9 also localized to them upon depletion of Myo2. We tagged Atg9 with 3 copies of mCherry in a strain that expressed PrD-GFP, depleted Myo2 and performed co-localization studies. As previously reported, it is not the entire pool of Atg9 that localizes to the PAS, but only a small subfraction, because Atg9 is present in various different Atg9-vesicle pools [47]. Consistent with this, we observed without depletion of Myo2 multiple foci of Atg9-3xmCherry, one of which localized adjacent to the single IPOD focus of PrD-GFP in 78% of the cells. After depletion of Myo2 for 6 hours, we found that 77% of the multiple PrD-GFP foci had at least one focus of Atg9-mCherry adjacent to it (Fig 5A). This suggested that the accumulations containing Atg8, preApe1 and PrD-GFP were in close proximity to Atg9. We note that consistent with the localization of Atg9 to different Atg9 vesicle pools, we observed many more Atg9 foci as compared to PrD-GFP ones. Since Atg9 is an integral membrane protein, the punctate structures containing it next to PrD-GFP, RFP-Atg8 and preApe1 likely contain membranes, possibly Atg9 vesicles [21]. Since PrD-GFP localized close to preApe1 that is known to associate with Atg9 vesicles via it’s receptor Atg19 and the adaptor Atg11 for recruitment to the PAS [43], we wondered whether any of those components was required for targeting of PrD-GFP to the IPOD? We deleted those genes in a strain with Gal-inducible PrD-GFP and tested for IPOD formation after 6 hours of induction with galactose. None of the deletion strains showed any visible impairment of PrD-GFP recruitment to a single focus typical for the IPOD (S8 Fig).

The faithful translocation of preApe1 into the vacuole involves SNARE proteins [48]. Sec18, which we found to bind to the PrD-fiber resin (S1 Table), is a SNARE disassembly chaperone required for several vesicular fusion events including homotypic vacuole fusion and autophagy [49,50]. We therefore depleted the essential SEC18 using again the aid-degron strategy [38] (Fig 5D) and monitored the distribution of PrD-GFP after induction with galactose in the presence of auxin. Very similar to the depletion of Myo2 and Tpm1/2, we observed a multiple PrD-GFP foci phenotype (Fig 5B, middle panel) in 83% of the cells (Fig 5C) that were still viable (Fig 5E). The accumulation of PrD-GFP in Sec18 depleted cells was reversible when auxin was washed out (Fig 5B, right panel, and 5C). Furthermore, the depletion did not only affect the localization of PrD-GFP, but also of mCherry-Atg8 (Fig 5F). These data suggest that efficient recruitment of PrD-GFP to the IPOD and preApe1 to the PAS involves vesicular transport, consistent with a function of Myo2 in vesicle transport along actin cables [51]. If that was true, Myo2 should localize to accumulating PrD-GFP transport intermediates in Sec18 depleted cells. To test this, we labeled Myo2 with 3xmCherry in a strain with Gal inducible PrD-GFP and allowed for auxin-based depletion of Sec18 for 6 hours. Myo2 was mostly diffusely distributed in control cells (Fig 5G, upper panel), but accumulated in multiple foci after depletion of Sec18 (Fig 5G, lower panel). 74% of the PrD-GFP foci co-localized with Myo2-3XmCherry, and the foci containing both proteins superimposed well. In conclusion, our data suggest that the two substrates PrD-GFP and preApe1 are linked to a similar vesicular transport machinery during their recruitment to the IPOD or PAS, respectively. Further support for the involvement of vesicular transport in PrD-GFP recruitment to the IPOD came from our observation about Sec14 and Sec21 that also bound to the immobilized PrD-GFP fibers (compare S1 Table). Sec14 is a phosphatidylcholine/phosphatidylinositol transfer protein that is involved in vesicular transport processes and is also required for autophagy [52]. Its depletion gave a similar phenotype of accumulation of multiple PrD-GFP foci as compared to Myo2 and Sec18. Ceasing depletion of Sec14 also restored the recruitment defect (S9A–S9C Fig). Furthermore, depletion of Sec21, a component of COPI vesicles that was also found on Vid vesicles that target specific proteins such as fructose-1,6-bisphosphatase (FBPase) for vacuolar degradation [53], gave a similar, reversible phenotype of multiple fluorescent PrD-GFP foci as compared to Myo2 or Sec18 depletion (S9D–S9F Fig).

### PrD-GFP is turned over only after liberation from the IPOD by Hsp104

**Fig 6:**
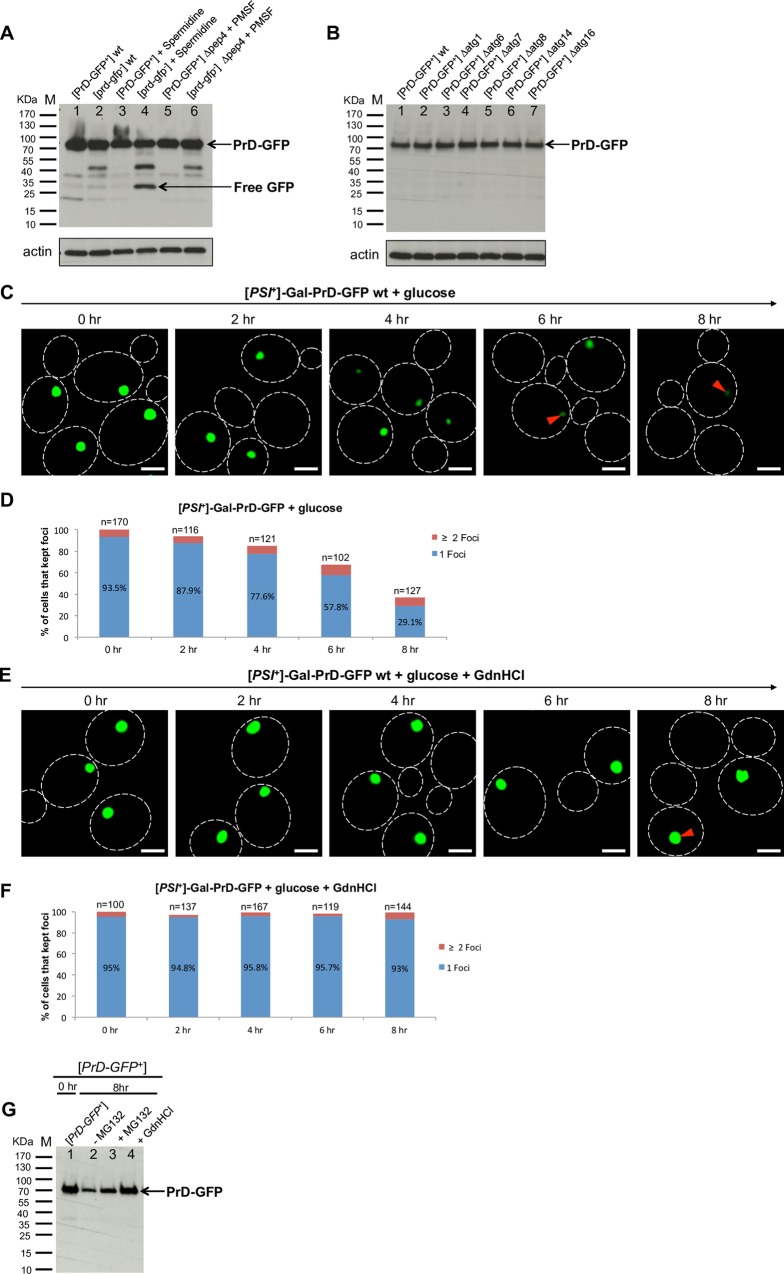
PrD-GFP can be turned over only after liberation from the IPOD by Hsp104. (**A**) Strains expressing PrD-GFP constitutively under control of the GPD promoter with a deletion in the endogenous prion domain in SUP35 were either in the prion state [*PrD-GFP*^+^] (RK3) or in the non-prion state [*prd-gfp*^-^] (RK4). The PEP4 gene was deleted as indicated. Strains were grown to saturation, diluted to an OD_600_ of 0.15 and were further incubated untreated or in the presence of 4 mM spermidine or 1 mM PMSF as indicated. After ~4 hours, cells were harvested, adjusted to identical cell numbers and subjected to Western Blot analysis using an anti-GFP antibody. Turnover of PrD-GFP can be judged by release of free GFP moiety. An antibody against actin was used as loading control. (**B**) Yeast strains as in (A) that propagated PrD-GFP in the prion state ([*PrD-GFP*^+^]) but carried a different deletion in autophagy-related genes as indicated were grown to logarithmic growth phase, adjusted to identical cell numbers and analyzed by Western Blotting with an anti-GFP antibody and an anti-actin antibody as loading control. (**C**) PrD-GFP was induced with galactose for 6 hours in a [*PSI*^+^] strain to allow deposition of PrD-GFP at the IPOD (RK1), pelleted and resuspended in YPD for further incubation. At indicated times, aliquots were withdrawn, the OD_600_ was determined prior to fixation and fluorescence microscopy. **(D)** IPOD decay was determined for the aliquots withdrawn in (C) by plotting of the number of cells with aggregates as % of cells with aggregates originally present in the culture at the shift to YPD (compare methods for details). **(E, F)** Same experiment than in (C, D), but after PrD-GFP induction, the cells were resuspended in YPD + 5 mM of GdnHCl (Hsp104 inhibition). **(G)** PrD-GFP was induced with galactose for 6 hours in a [*PSI*^+^] strain (RK1 *Δpdr5*) to allow for deposition of PrD-GFP at the IPOD, pelleted and resuspended in YPD. At "time 0h", an aliquot for determination of PrD-GFP levels by Western Blotting was withdrawn. Subsequently, the culture was split into three cultures. One was left untreated (- MG132), MG132 (80 μM) was added to the second (+ MG132) and refreshed every 2 hours, and GdnHCl to the third one (+ GdnHCl). After 8 h of further incubation, the same volume of culture as for "time 0h" (compare methods) was withdrawn and analyzed by Western Blotting with an antibody against GFP.

Since PrD-GFP aggregates use a similar recruitment machinery as compared to substrates like preApe1 to the PAS, it is not surprising that the IPOD localizes in close proximity to it. At the PAS, preApe1 complexes are enwrapped into CVT vesicles that subsequently fuse with the vacuole for delivery of their content into the lumen [46]. Therefore, we asked if amyloid aggregates are recruited close to the PAS to facilitate direct delivery to the vacuole for possible autophagic turnover in a similar mechanism? To test this, we used a strain that expresses PrD-GFP constitutively and deposits the corresponding aggregates at the IPOD [15]. We reasoned that if there is significant turnover of PrD-GFP IPODs by autophagy, then inhibition of autophagy should increase the steady state levels of PrD-GFP, whereas additional induction of autophagy should decrease them. Thus we compared the levels of PrD-GFP by Western Blotting in a strain that was left untreated (Fig 6A, lane 1) with a strain where autophagy was induced by spermidine [54] (Fig 6A, lane 3) or inhibited by 1 mM of PMSF in a *pep4Δ* strain background [55] (Fig 6A, lane 5). As internal control, we also included a strain with PrD-GFP in the soluble non-prion state, and treated it in the same way (Fig 6A, lanes 2, 4, 6). As seen in Fig 6A, the amounts of PrD-GFP were in all samples very similar. The only slight difference was seen after induction of autophagy in the strain with PrD-GFP in the soluble state (Fig 6A, lane 4). Here, we observed a band of free GFP emerging (Fig 6A, lane 4). This sample also served as our internal control for successful induction of autophagy and subsequent turnover of PrD-GFP, because induction of autophagy leads to enclosure of random parts of the cytoplasm, including some of the soluble PrD-GFP, into autophagosomes followed by turnover in the vacuole [21]. GFP is rather stable in the vacuole, which leads transiently to free GFP when GFP fusion proteins are degraded [56]. In contrast to the strain with soluble PrD-GFP, we did not observe any significant band of free GFP when PrD-GFP was present at the IPOD (Fig 6A, lane 3). Furthermore, inhibition of autophagy did not lead to markedly increased PrD-GFP levels in any of our strains. In a second set of experiments, we tested different mutants defective for autophagy [55], but the levels of PrD-GFP were indistinguishable from the wild type control and no bands of free GFP were detected (Fig 6B). Furthermore, no striking difference in the intensity or morphology of PrD-GFP at the IPOD was detected in those strains by fluorescence microscopy (S10A Fig). Taken together, in contrast to what was for example suggested for aggresomes in mammalian cells [7,57], PrD-GFP at the IPOD is not efficiently turned over in bulk by autophagy.

We had observed earlier that the IPOD depositions decayed progressively, but very slowly over time (S4A Fig). These data hinted that PrD-GFP aggregates present at the IPOD are processed, but more gradually rather than in bulk. A well-characterized factor known to process and remodel prion aggregates is Hsp104 [58–61]. To test whether PrD-GFP at the IPOD is subject to Hsp104 dependent processing, we induced PrD-GFP expression by galactose for 6 hours before we shifted the culture to glucose to stop further expression of PrD-GFP. Then, we split the culture into two halves and further incubated the cells in the absence or presence of low concentrations of GdnHCl known to inhibit Hsp104 [45], and followed the pre-existing IPODs over time. We observed that the number of cells containing PrD-GFP at the IPOD as well as the amount of PrD-GFP in the residual IPODs decreased very slowly over time and several cell divisions (Fig 6C and 6D). In contrast, neither the number of cells with an IPOD nor the intensity of the PrD-GFP fluorescence at the IPOD changed visibly when Hsp104 was inhibited (Fig 6E and 6F). We note that when we determined the percentage of cells with aggregates before and after the glucose shift, we did not consider freshly budded cells emerging during the chase time with glucose, but only cells that were present before the chase (see methods for details). To reveal the possible fate of PrD-GFP extracted from the IPOD by Hsp104, we tested if the protein may be degraded by the proteasome by using the proteasomal inhibitor MG132. As a control for successful inhibition of the proteasome, we also monitored the decay of the known proteasomal substrate tGnd1-GFP [11] under identical conditions with and without addition of MG132 (S10B Fig). Thus we repeated the PrD-GFP decay experiment described above in a strain that was identical to the one used above, but carried additionally a deletion of the PDR5 gene to prevent export of MG132 out of the cells. We divided the culture after Gal-induction of PrD-GFP into 3 aliquots and shifted to glucose. While the first aliquot was left further untreated, we inhibited the proteasome by MG132 in the second one and Hsp104 with GdnHCl in the third one. After 8 hours in glucose, we monitored the levels of PrD-GFP by Western Blotting and compared them to the level at the beginning of the glucose chase (Fig 6G, lane 1). The amount of PrD-GFP was decreased in the control after the glucose chase (Fig 6G, lane 2). After inhibition of the proteasome, the decrease in PrD-GFP was much less pronounced (Fig 6G, lane 3). After inhibition of Hsp104 by GdnHCl, the amount of PrD-GFP was identical to that in the beginning of the glucose chase (Fig 6G, lanes 1 and 4). Thus PrD-GFP can only be turned over after Hsp104 mediated extraction from the IPOD. This was consistent with the fact that the number and overall intensity of the IPODs did not change after inhibition of Hsp104 (Fig 6E and 6F). Thus proteasomal inhibition reduced the turnover of PrD-GFP after liberation by Hsp104, which demonstrates an involvement of the proteasome in turnover of PrD-GFP, but it did not block turnover completely. Therefore, it is well possible that some of the extracted PrD-GFP is also turned over by other pathways, e.g. autophagy [62]. However, the kinetics of IPOD decay by Hsp104 were probably too slow to detect such possible turnover under steady state conditions (Fig 6A). Future quantitative studies would be required to reveal the detailed fate of PrD-GFP and the single contributions of different cellular turnover pathways.

In summary, these results suggest that PrD-GFP deposition at the IPOD may serve a temporary storage or sequestration function when the aggregate load exceeds the capacity of the proteolytic machineries involved in aggregate turn over.

### Model for recruitment of amyloid substrates to the IPOD

**Fig 7:**
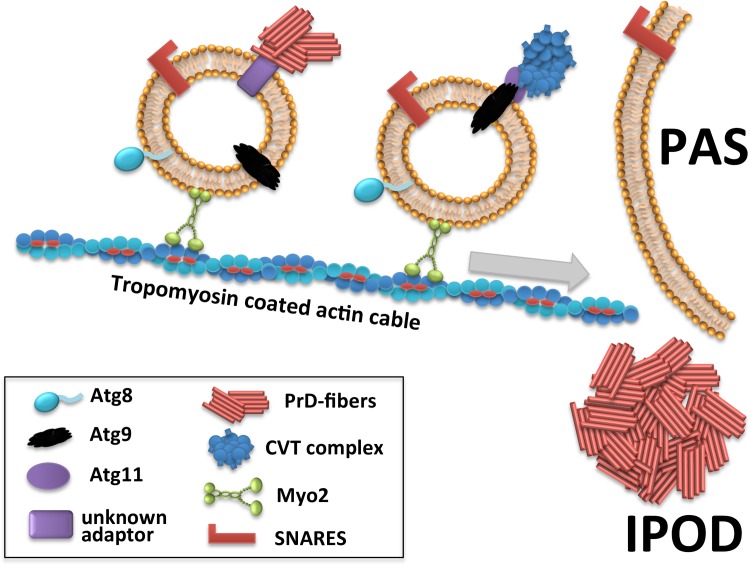
Model for recruitment of PrD-GFP prion aggregates to the IPOD.

We observed that the depletion of Tpm1/2, Myo2 and Sec18 does not affect the integrity of the IPOD once it has formed. Therefore, we propose the model (Fig 7) that the depletion of these proteins interferes with the recruitment of the studied substrates to one particular site in the cell where an IPOD will then become visible. Currently, no specific structural components of the IPOD deposition site are known. Hence it is not clear if the IPOD pre-exists even in the absence of substrates, or if it only forms once the newly discovered recruitment machinery directs substrates to a unique perivacuolar site near the PAS where it will then form. The latter possibility would parallel mammalian aggresomes that form at the MTOC because this is where the microtubules, along which the aggregates are transported, guide them to [63]. In our model, PrD-GFP associates with a vesicular transport machinery that employs Myo2 and tropomyosin coated actin cables to recruit PrD-GFP aggregates and other amyloid aggregates to a PAS adjacent localization termed IPOD. Interestingly, CVT pathway components destined for recruitment to the PAS use a similar recruitment machinery. Myo2 was identified here as a linking factor between this vesicular recruitment machinery and the actin cytoskeleton.

## Discussion

### PrD-GFP and preApe1 are recruited via a similar machinery to the adjacent IPOD and PAS, respectively

We investigated the mechanism of prion amyloid aggregate deposition at the IPOD, which is located adjacent to the PAS [10,15] where the cell initiates formation of autophagosomes and CVT vesicles [21,22]. Unexpectedly, the recruitment of prion aggregates utilized a similar machinery as compared to proteins destined for the PAS.

The CVT pathway delivers vacuolar precursor peptidases including preApe1 to the PAS where they are enclosed into CVT-vesicles that subsequently fuse with the vacuolar membrane to deliver their content into the lumen. For its recruitment to the PAS, preApe1 forms large multimeric complexes that are loaded onto Atg9 vesicles via the specific receptor Atg19 and the adaptor protein Atg11 [22,46]. Since Atg9 vesicles and preApe1 failed to reach the PAS after impairment of the integrity of the actin cytoskeleton or the Arp2/3 complex [42,64], it was proposed that Atg9 vesicles loaded with the preApre1 complex are moved towards the PAS either directly along actin cables or indirectly by inducing actin nucleation through recruitment of Arp2/3 [43]. In those studies however, it remained unclear what factor present at the Atg9 vesicles tethers them to the actin cables. Our finding that depletion of Myo2 leads to a failure of preApe1 recruitment to the PAS would favor a direct Myo2 mediated movement of Atg9 vesicles along actin cables (compare Fig 7), similar to the known Myo2 based transport of vacuole-derived vesicles along actin cables from the mother into the bud during vacuole inheritance [65]. Our observation that the depletion of the SNARE chaperone Sec18 [66] also results in disruption of preApe1 delivery to the PAS, is in full agreement with the recent findings that impairment of SNARE proteins interferes with the CVT pathway and proper recruitment of structural components to the PAS [48].

The reversible co-accumulation of PrD-GFP with CVT pathway components upon impairment of the recruitment machinery led us to the hypothesis that amyloid aggregates and precursor peptidase complexes are transported by a similar vesicular machinery to the adjacent destination sites IPOD and PAS, respectively (Fig 7). It remains currently unclear whether PrD-GFP aggregates and preApe1 complexes are loaded to identical vesicles or to different types of vesicles. However, both classes of substrates can be recruited independently of each other e.g. PrD-GFP in the absence of preApe1 and vice versa, and both require Myo2. We did observe slight differences for the co-localization of different CVT pathway components with PrD-GFP. For example Atg8 was a little bit more abundantly detected at PrD-GFP accumulations as compared to preApe1. Furthermore, accumulations of PrD-GFP superimposed better with those of RFP-Atg8 and Myo2-3XmCherry as compared to preApe1 and Atg9. This may hint to the possibility that PrD-GFP and preApe1 use similar, but not identical vesicles for their recruitment, and those vesicles accumulate in the same cellular locations upon impairment of either SNARE mediated vesicular transport or actin cable based transport. However, the differences could also be of technical nature, for example because fluorescently tagged components with lower abundance may have a different detection threshold as compared to more abundant ones, or because the recruitment machinery was overloaded and could not bind the entire fractions of PrD-GFP and preApre1, especially when the two substrates were expressed at the same time.

The association of amyloid aggregates with the actin cytoskeleton has been reported before by different labs [28–30,67]. Strikingly, a partial co-localization of Htt103Q aggregates with Myo2 and Sec18 was previously observed during their asymmetric partitioning during cell division [29]. However, those aggregates were not concentrated in one central IPOD like inclusion, but present in multiple dispersed structures. Whether they represent targeting intermediates destined for IPOD-like deposition sites that failed to be properly deposited for unknown reasons, or are different independent structures, remains to be elucidated. Nevertheless, our observation that the additional IPOD substrates [10] Htt103Q-CFP, Rnq1-GFP and Ure2-YFP can also not be recruited to the IPOD properly after depletion of Myo2 function suggests that the recruitment machinery to the IPOD discovered here is used by different amyloids.

### Tethering of prion aggregates to the CVT recruitment machinery

A remaining open question is what the linking factor between prion aggregates and the vesicular recruitment machinery could be. The deletion of Atg19, which is the receptor for large preApe1 complexes [22,46], did not effect proper localization of PrD-GFP to a single large IPOD-like deposition, suggesting that PrD-GFP is not recognized by the same receptor as preApe1.

Hsp104 and the stress inducible protein Lsb2 were previously suggested to link different types of aggregates including amyloids/prions with the actin cytoskeleton [28,40]. Although we don’t exclude the possibility that these 2 proteins contribute to a tethering function in the recruitment machinery discovered here, we consider the impact for such a function less crucial for the following reasons. Lsb2 is present in minor amounts in unstressed cells [28], therefore we did not expect it to be abundant enough in our experiments performed at standard, non-stress conditions. With regard to Hsp104 as a possible tether, it was observed that the IPOD stays intact and can become even bigger in size upon time in cells where Hsp104 is inhibited [15,23]. Thus, Hsp104 function seems at least not essential for ongoing recruitment of prion aggregates to the IPOD.

Alternatively, PrD-GFP could be bound by structural components of the vesicular transport machinery directly. In favor of this, the endocytosis machinery that shares some of the Sec components also involved in autophagy/CVT pathway [48], is also involved in initiating aggregation of proteins with extended polyglutamine stretches [68]. Moreover, Sla1 and Sla2, also components of the endocytosis machinery, have previously been implicated in [*PSI*^*+*^] prion aggregate handling [25,26]. Finally, amyloid aggregates and prefibrilar oligomers are also known to have an intrinsic affinity to bind directly to lipid membranes [69], which leaves the possibility that direct lipid interactions with PrD-GFP aggregates might be involved in the tethering.

### Turnover of PrD-GFP deposited at the IPOD requires liberation by the Hsp104 disaggregation machinery

We observed that PrD-GFP uses a similar recruitment machinery as compared to CVT substrates to the PAS where they are subsequently enwrapped into autophagy related vesicles for delivery into the vacuolar lumen [46]. Therefore we wondered whether this recruitment of PrD-GFP to a PAS-adjacent site may serve the purpose to accumulate the aggregates at the PAS for autophagic turnover, similar to mammalian cells where aggresomes are believed to be engulfed by double membranes to form autophagosomes that subsequently can fuse with lysosomes for proteolytic degradation [63]. Interestingly, we did not find any positive hint for bulk turnover by autophagy. It is possible however, that at lower expression levels and hence lower aggregate load, amyloids could successfully be incorporated into autophagic vesicles, but that the capacity of this system can get overwhelmed and impaired by excess amyloid aggregates accumulating at the PAS. If this were the case, then the process of phagophore formation would be impaired at an early stage, as previous electron microscopy studies that imaged in great detail the [*PSI*^*+*^] aggregate depositions at the IPOD never observed any recognizable membrane structure resembling for example remnants of autophagosomes [13–15]. Alternatively, amyloids might be recognized as potential substrates for turnover by autophagy, but cannot be processed by the corresponding machinery for unknown reasons, even at lower expression levels of the prion determining protein. As a third possibility, accumulation of PrD-GFP at the IPOD could serve a temporary storage function of excess PrD-GFP when the capacity of downstream proteolytic machineries involved in degradation is not sufficient. This storage would sequester the amyloid aggregates, possibly to avoid harmful effects of the aggregates [20] until processing factors such as Hsp104 and possible downstream machineries are available. Either way, once accumulating at the IPOD, amyloid aggregates can very slowly be extracted by the Hsp104 disaggregation machinery and may subsequently be subjected to proteolysis by either the proteasome [30] or autophagy [62]. Furthermore, deposition of aggregates at the IPOD was also suggested to facilitate asymmetric aggregate inheritance [10,15,28,70,71].

## Materials and Methods

### Chemicals and antibodies

Yeast cultures were grown in rich medium YPD (BD Difco 1% yeast extract, 2% peptone and 2% glucose) or YPG (BD Difco 1% yeast extract, 2% peptone and 2% galactose). Standard synthetic media (0.17% yeast nitrogen base without amino acids and ammonium sulfate [YNB w/o aa and as], 2% glucose, 0.07% CSM, 0.5% ammonium sulfate) lacking particular amino acid was used to select the yeast transformants. Ni-NTA (Novagen) was used for purification of biotin-labeled PrD protein. D-biotin (Roche) was used for biotinylation of PrD. Streptavidin Dyna Beads were purchased from Hyglos. Indole-3-acetic acid sodium salt (IAA) was bought from Sigma. Anti- HA antibody was purchased from Sigma. Anti-GFP antibody was purchased from Roche. The anti-actin antibody was bought from Millipore.

### Plasmids, yeast strains, growth conditions and yeast standard methods

Plasmids used in this study are listed in S3 Table. pH10sumo-PrD-TEV-Avi, used to synthesize biotinylated PrD, was generated by fusion PCR from “pH10sumo” [72] using the primers P1: 5’-GTGAGCGGATAACAATTCCCCTC and P2: 3’-CCTTGGTTTGAATCCGACATaccaccaatctgttctctgtgagcctcaataatatcg to amplify the N-terminal His-sumo-tag and the primers P3: gaggctcacagagaacagattggtggtATGTCGGATTCAAACCAAGGCAACAATC and P4: ggtacccgGGATCCATCGTTAACAACTTCGTCATCC to amplify PrD-GFP [73]. The amplification product was then cloned into a Avi-tag (Avidity Avitag) containing construct [74]. pHis-Sumo-PrD-STOP was generated from pH10sumo-PrD-TEV-Avi by introducing a STOP codon by quickchange PCR. Yeast strains used in this study are derivatives of 74D-694 [58] that expressed PrD-GFP integrated into the genome from the galactose inducible promoter [73] (termed 74D [*PSI*^*+*^]-Gal-PrD-GFP) or derivatives of 74D-694 that contained a deletion of the PrD in the endogenous SUP35 locus and expressed PrD-GFP integrated into the genome from the constitutive GPD promoter [15] (termed 74D-GPD-PrD-GFP). Additional genetic manipulations performed in these strains are listed in the S2 Table. Yeast cells were grown in YPD or synthetic drop-out media. Antibiotics, if needed, were added as indicated.

SDS-PAGE and Coomassie staining was performed according to standard methods, Western Blotting was performed using a standard tank blot system (BioRad). Paraformaldehyd (PFA) fixation of yeast cells was performed by adding equal volumes of 8% PFA in PBS to a yeast culture (final PFA concentration 4%) and incubation for 10 min at room temperature, followed by washing with PBS.

### Purification of 10xHis-Sumo-PrD-Avitag and 10xHis-Sumo-PrD-STOP

The two constructs 10xHis-Sumo-PrD-Avitag and 10xHis-Sumo-PrD were transformed into BL21 (DE3) harboring a plasmid coding for biotin ligase (chloramphenicol resistance) and a pre-culture was grown over night at 37°C. 1.5 liters of Terrific Broth (TB) + ampicillin and chloramphenicol were inoculated with 2% of the pre-culture and grown to an OD_600_ of 0.7 to 0.8. Then expression was induced for 4 h with 1 mM IPTG in the presence of 20 mg/l of biotin in the media at 37°C. Cells were harvested by centrifugation, resuspended in 20 ml of lysis buffer (40 mM HEPES-KOH pH 7.4, 150 mM KCl, 5 mM MgCl2, 5% glycerol, 10 mM imidazole, 2 mM ß-mercapto-ethanol, protease inhibitors) and lyzed by a combination of French press and sonication. After centrifugation (45 min, 12.000 rpm) to remove insoluble material, the supernatant was added to 1.5 ml of Ni-NTA resin (Novagen) equilibrated in lysis buffer and rotated gently at 4°C for 2 h. The beads were washed 3 times in lysis buffer, transferred to a column and eluted in 10–15 fractions with 250 mM imidazole. The eluted protein was partially denatured with urea at a final concentration of 2M. Ulp1 was then added at 4 μg/μl to cleave the sumo tag and the protein was dialyzed against 3 l of lysis buffer + 2M urea at 4°C over night. To remove the cleaved His-sumo tag, the protein was again subjected to Ni-NTA for 1 hour prior to concentration to roughly 6 mg/ml of PrD using 30 kDa MWCO amicon concentrators. The concentrated solution was then brought to 8M urea and the protein was methanol precipitated to further concentrate by a factor of 10 and stored in 6 M of GdnHCl at –80°C.

### *In vitro* formation of PrD-fibers and their immobilization to a streptavidin resin

Purified PrD-Avitag (in GdnHCl) was mixed 1:50 with PrD-STOP (in GdnHCl) and the mixture was diluted 1:100 into fiber formation buffer (5 mM K/KHPO4, pH 7.4, 150 mM NaCl) and rotated at 8 rpm over night at room temperature. Formed fibers were fragmented by sonication for 5 min a water bath sonicator. Subsequently, the fibers were pelleted and resuspended into equal volumes of fiber attachment buffer (25 mM Tris/HCl, pH 7,4, 150 mM KAc, 5 mM MgAc, 5% glycerol, 1 mM DTT, 1 mM PMSF, protease inhibitors)

To immobilize the PrD fibers to avidin coated magnetic beads (hyglos), the volume equivalent to 50 μl of beads pellet (use magnet) was incubated with 5 mg/ml of BSA in PBS overnight in the cold room. Subsequently, the beads pellet was equilibrated 1 x with 500 μl of 5 mg/ml BSA in fiber attachment buffer for 1 hour and washed two times with 500 μl of fiber attachment buffer without BSA. In parallel, 375 μl of fibers/50 μl of beads pellet were washed twice in fiber attachment buffer and then incubated with the avidin coated magnetic beads for ~ 1–2 hours at 4°C. Unbound fibers were removed by washing the beads 3 x carefully with 200 μl of fiber attachment buffer.

### Yeast cell lysate preparation in a mixer mill

50 ml of a yeast culture in logarithmic growth phase at an OD_600_ ~ 0.4–0.6 were harvested and the pellet was transferred to 1.5 ml eppendorf tubes, resuspended in 100 μl of fiber attachment buffer (25 mM Tris/HCl, pH 7.4, 150 mM KAc, 5 mM MgAc, 5% glycerol, 1 mM DTT, 1 mM PMSF, protease inhibitors) and dripped in liquid nitrogen present in a 2-ml round bottom eppendorf tube that contained a 7 mm stainless steel ball. After boiling out of the liquid nitrogen, the tubes were closed and placed in an adaptor for 2 ml tubes into a Retsch Mixer Mill MM 400 and agitated for 2 x 2 min at 30 Hz. The sample was cooled in liquid nitrogen in between the two rounds of agitation. The resulting powder of lysed cells was transferred into a 1.5 ml tube and resuspended into 500 μl of fiber attachment buffer, spun at 500 g and 4°C to remove cell debris, followed by a second spin at 14000 g for 30 min to separate insoluble from soluble cellular components. The soluble fraction was used for the fishing experiment.

### Identification of amyloid binding proteins

A 50 μl-volume of avidin coated magnetic beads containing immobilized biotinylated PrD fibers was incubated with 500 μl of yeast cell lysate (~2 mg/ml protein concentration) in low binding eppendorf tubes at 4°C over night under gentle agitation in an overhead incubator. Subsequently, the beads were washed 4 x with 200 μl fiber attachment buffer before the proteins bound to PrD-GFP were eluted by incubating at 95°C in SDS-PAGE sample buffer (Laemmli buffer) for 10 min.

### In-gel tryptic digestion and LC-MS/MS analysis

After SDS-PAGE, Coomassie stained bands were cut out with a scalpel and processed as described previously [75]. In brief, samples were reduced, alkylated and digested with trypsin. Peptides were extracted from the gel pieces, concentrated in a speedVac vacuum centrifuge and diluted to a total volume of 30 μl with 0.1% TFA. 25 μl of the sample was analyzed by a nanoHPLC system (nanoAcquity, Waters) coupled to an ESI LTQ Orbitrap XL mass spectrometer (Thermo Fisher). Sample was loaded on a C18 trapping column and separated on an analytical column (75μm x 250mm) with a flow rate of 300nl/min in an acetonitrile-gradient (3%-40%). One survey scan (res: 60000) was followed by 5 information dependent product ion scans in the ion trap. The uninterrupted MS/MS spectra were searched against “The Swissprot_2014_04 *Saccharomyces Cerevisiae* Database”.

### Fluorescence microscopy

Cells were generally grown in liquid culture to mid-log phase, fixed with 4% of paraformaldehyde (PFA), resuspended in PBS and examined with an Olympus IX81 inverted microscope with a 100x/1.45 oil objective and narrow band-pass filters for co-localization studies with different fluorescent proteins at room temperature. Images were taken with a Hamamatsu ORCA-R2 camera in the Olympus Excellence Software. Unless indicated differently, z-stacks of cells with a step width of 0.2 μm were taken and the single layers were merged as maximum intensity projection into 1 image. Images were analyzed in ImageJ and brightness and contrast were linearly adjusted. All fluorescence images shown in the manuscript, including time-lapse microscopy images and videos, were de-blurred using the Wiener Filter deconvolution algorithm present in the Olympus Xcellence software and then merged into one image.

### Time lapse microscopy

Time-lapse microscopy was performed on agarose pads of 20 x 20 x 1 mm, prepared by pouring ultrapure agarose (2% w/v) in SD or YPD media directly onto a microscope slide. After addition of the cells, the pad was covered with a cover slide and sealed with melted VLAP wax (1:1:1 Vaseline:lanolin:paraffin). Every 2–5 min, we collected a stack of ~15 optical sections spaced 0.2–0.3 μm apart.

### PrD-GFP decay experiments

To monitor the decay of pre-existing PrD-GFP single foci (IPODs), we used cells where the PrD-GFP IPOD was pre-formed for 6 hours by galactose induction. The cells were then shifted to glucose-based media to stop synthesis of PrD-GFP, diluted to an OD_600_ of 0.3 (“time point 0”) and further incubated for up to 8 hours at 30°C. During this time, the cells grew further. We withdrew samples at the indicated time points, measured the OD_600_ to monitor how often the cells had divided in the glucose-based media, fixed the cells and counted the number of total cells as well as the number of cells that still had visible PrD-GFP aggregates. This was done for roughly hundred cells per 0.3 OD_600_ units. Finally, based upon the measured OD_600_ for the time point of interest, we calculated the number of the newly born cells since the beginning of the shift to glucose (OD at “time point 0” was 0.3) and subtracted this number from the total number of cells determined. Finally, we determined how many of those cells present since the beginning of the decay experiment still had a PrD-GFP aggregate (in %). This method is based upon the observation that the IPOD is retained in the mother cells during cell divisions [15,70].

When the amount of PrD-GFP was determined after such a decay experiment in glucose by Western Blotting (e.g. Fig 6G), we did not use the same number of cells for Western Blotting, but the same volume of culture, because the cells sometimes grew at different rates. Since new synthesis of PrD-GFP from the Gal promoter was ceased by glucose, the same volume of culture should contain the same amount of original PrD-GFP that may have been partitioned between a mother and multiple progeny or stayed in fewer cells when cells divided slower. For these reasons, no classical loading control could be included for this Western Blot.

### Quantification of different aggregation phenotypes

For determination of the phenotype of multiple dispersed accumulations of PrD-GFP or various PAS substrates versus one single central aggregate at the IPOD or PAS, respectively, the number of cells with one single accumulation/aggregate versus more than one accumulation/aggregate was determined from merged Z-stacks and plotted as “percentage”. The degree of co-localization of PrD-GFP with various other proteins including preApe1, Atg8, Atg9 or Myo2 was determined from merged z-stacks. All clearly distinguishable PrD-GFP fluorescent foci were counted. Subsequently, it was analyzed how many of those PrD-GFP accumulations co-localized at least partially with the corresponding other protein. It is given as “percentage of co-localization” in the figure legends.

## Supporting Information

S1 Fig Co-localization of Tpm1/2 with PrD-GFP. **(A)** Co-localization of mCherry fusions of tpm1/2 with PrD-GFP in 74D-694-ΔPrD (SUP35) [*PrD-GFP*^+^] strain (RK3-TPM1/2-mCh). Logarithmic growth phase culture was fixed and analyzed by fluorescence microscopy as described in methods. After deconvolution, a merged image of the z-stacks taken in the GFP- and the mCherry channels, respectively, were overlaid. Scale bar = 2 μm.
(PDF)

S2 Fig Effects of long term depletion of Tpm2, Myo2 and Sec18. (**A**) Wild type (wt), *tpm1Δ* Tpm2-aid, Myo2-aid and Sec18-aid strains (RK1, RK1c, RK1e, RK1g) were streaked onto SD-ura-leu plates containing 5 mM auxin and incubated for 2 days at 30° C. **(B)** Western blot analysis of a Myo2-aid strain (RK1e) without depletion (- Aux) or after auxin-based depletion (+ Aux) for 6 hours. Myo2 was detected with an antibody against an HA-tag present in the aid-degron-tag. An anti-actin antibody was used as loading control. **(C)** Quantitative determination of cell viability by Colony-Forming Units (CFU) Assay upon Tpm1/2, Myo2, and Sec18 depletion. Percentage viability of a [*PSI*^+^] strains with different C-terminal aid-tag in MYO2, TPM1/2 and SEC18 and the [*PSI*^+^] wild type strain (wt). PrD-GFP was induced with galactose for 6 hours in the absence or presence of 20 mM auxin. After 6 hrs of depletion, cultures were serially diluted in YPD from OD_600_ of 1.0 and 100 μl from the last two dilutions (10^−4^ and 10^−5^) were spread on YPD plates and grown at 30°C for 2 days for counting the resulting colonies formed by viable cells. The data are represented as means S.D. of three independent biological replicates. (**D**) Corresponding pictures of colonies formed by viable cells as in C.
(PDF)

S3 Fig Rescue of Myo2 and Tpm1/2 function by auxin wash-out causes re-fusion of PrD-GFP to 1 single IPOD. **(A)** Movie showing refusion of multiple PrD-GFP foci into one single IPOD upon washout of auxin. PrD-GFP was induced with galactose for 6 hours in the presence of 20 mM auxin in a [*PSI*^+^] Myo2-aid strain (RK1e). Subsequently, cells were pelleted, resuspended in YPD (glucose chase) and placed onto a microscope slide with a little agarose pad for time-lapse microscopy. Z-stacks with a step width of 0.3 μm were acquired every 2–5 min during one hour. Images were subsequently deblurred using the Wiener Filter algorithm, merged into 1 layer and combined into a movie. **(B)** PrD-GFP was induced with galactose for 6 hours in a [*PSI*^+^] strain containing a deletion of Tpm1 and a C-terminal aid tag in Tpm2 (RK1c). Subsequently, cells were pelleted, resuspended in YPD without auxin to rescue Tpm1/2 function and further incubated for 60 min prior to fixation and microscopic analysis. Subsequently, PrD-GFP localization in either 1 or more than 1 foci was determined and plotted as % in the absence or presence of 20 mM auxin or after auxin washout (1 hr recovery).
(PDF)

S4 Fig Myo2 depletion impairs recruitment of PrD-GFP to the IPOD rather than IPOD integrity. **(A)** Fluorescence microscopy images for the experiment shown in Fig 3E: PrD-GFP was induced with galactose for 6 hours in the absence of auxin in a [*PSI*^+^] Myo2-aid strain (RK1e), cells were pelleted and resuspended in YPD media in the presence of 20 mM auxin. Aliquots were withdrawn every 2 hours, fixed and analyzed by fluorescence microscopy. **(B)** Fluorescence microscopy images for the experiment shown in Fig 3F: PrD-GFP was induced with galactose in the presence of 20 mM auxin in a [*PSI*^+^] Myo2-aid strain (RK1e). After the indicated times, aliquots were withdrawn, fixed and analyzed by fluorescence microscopy. Scale bar = 2μm.
(PDF)

S5 Fig Myo2 is also essential for proper accumulation of different amyloid substrates (Rnq1, Ure2 and Htt103Q) at the IPOD. (**A, C**) Rnq1-GFP was induced with galactose for 6 hours in a [*PSI*^+^] strain with a C-terminal aid-tag in MYO2 (RK5b Rnq1-GFP) (left and middle panel) (A) or a wild type (wt) strain (RK6) (C), in the absence or presence of 20 mM of auxin as indicated. Subsequently, cells were pelleted, resuspended in YPD (glucose chase) without auxin to restore Myo2 function and incubated further for 60 min (auxin washout, right panel, (A)) prior to fixation and fluorescence microscopy. (**B, D**) Quantification of Rnq1-GFP foci upon depletion of Myo2 (B) or in the wild type (wt) (D). Frequencies of cells with 1 single focus or more than 1 foci are given in %. (**E, G**) Same experiment as in (A), but in a [*PSI*^+^] Myo2-aid strain (E) or a wild type strain (G) with a Ure2-YFP construct integrated into the genome under control of the Gal1 promoter (RK5b Ure2-YFP, RK7) (**F, H**) Quantification as in (B and D), but in a [*PSI*^+^] Myo2-aid strain with Ure2-YFP integrated into the genome under control of the Gal1 promoter. (**I, K**) Same experiment as in (A), but in a [*PSI*^+^] Myo2-aid strain or a wild type (wt) strain with a Htt103Q-CFP construct integrated into the genome under control of the Gal1 promoter (RK5b 103Q-CFP, RK8) (**J, L**) Quantification as in (B and D), but in a [*PSI*^+^] Myo2-aid strain with a Htt103Q-CFP construct integrated into the genome under control of the Gal1 promoter. Scale bar = 2 μm.
(PDF)

S6 Fig Depletion of Myo2 impairs proper recruitment of substrates to the IPOD, and restoration of Myo2 function leads to recruitment of different substrates to the same single IPOD site. **(A)** Rnq1-GFP was induced with galactose for 6 hours in the absence of auxin in a [*PSI*^+^] Myo2-aid strain (RK5b Rnq1-GFP), cells were pelleted and resuspended in YPD media in the presence of 20 mM auxin. Aliquots were withdrawn every 2 hours, fixed and analyzed by fluorescence microscopy. (**B**) Quantification of Rnq1-GFP foci that corresponds to S6A Fig. Rnq1-GFP localization in either 1 or more than 1 foci was determined from cells that still carried Rnq1-GFP aggregates and plotted as %. (**C**) Co-localization of Ure2-YFP with Htt103Q-CFP. Both Ure2-YFP and Htt103Q-CFP were induced with galactose for 6 hours in the absence or presence of 20 mM auxin in a [*PSI*^+^] Myo2-aid strain (RK9) (upper and middle panel). Subsequently, cells were pelleted, resuspended in YPD without auxin to restore Myo2 function and incubated further for 60 min (auxin washout, bottom panel) prior to fixation and fluorescence microscopy. Co-localization:–auxin, +auxin and auxin washout: 100%, n = 100–150 foci. Scale bar = 2 μm.
(PDF)

S7 Fig Washout of auxin causes refusion of multiple GFP-Atg8 foci to one single focus. **(A)** Expression of GFP-Atg8 was induced with galactose for 6 hours in the presence of 20 mM auxin in a [*PSI*^+^] Myo2-aid strain containing an N-terminal genomic GFP-fusion to Atg8 under control of the Gal promoter (RK5f). Cells were pelleted, resuspended in YPD in the absence of auxin to restore Myo2 function and placed onto a microscope slide with an agarose pad for time-lapse microscopy. Z-stacks with a step width of 0.2 μm were acquired every 4 min. Images were deblurred using the Wiener Filter algorithm and combined into one image. **(B)** Movie showing re-fusion of GFP-Atg8 foci generated during Myo2 depletion and subsequent washout of auxin as described in (A). **(C)** Quantification of GFP-Atg8 localization in either 1 or more than 1 foci in % in the absence or presence of 20 mM auxin or after auxin washout (1 hr recovery). Scale bar = 2μm.
(PDF)

S8 Fig Neither CVT pathway substrates (preApe1 and Ams1) nor different CVT components (Atg9, Atg11 or Atg19) are essential for PrD-GFP recruitment to the IPOD. PrD-GFP was induced with galactose for 6 hours in a [*PSI*^+^] wt strain or an identical strain, but with the indicated deletions. Cells were fixed and analyzed by fluorescence microscopy. Scale bar = 2μm.
(PDF)

S9 Fig Effect of Sec14 and Sec21 depletion on accumulation of PrD-GFP at the IPOD. (**A**) PrD-GFP was induced with galactose for 6 hours in the absence or presence of 20 mM auxin (left and middle panel) in a [*PSI*^+^] strain with a C-terminal aid-tag in SEC14 (RK1h). Subsequently, cells were pelleted, resuspended in YPD (glucose chase) without auxin to restore Sec14 function and incubated further for 60 min (auxin washout, right panel) prior to fixation and fluorescence microscopy. (**B**) Quantification of PrD-GFP foci upon depletion of Sec14. Frequencies of cells with 1 single focus or more than 1 foci are given in %. Scale bar = 2 μm. (**C, F**) Western blot analysis of a [*PSI*^+^] Sec14-aid and Sec21-aid strain without (-Aux) or after depletion (+Aux) of Sec14 and Sec21 with an antibody against an HA-tag present in the aid-tag. An anti-actin antibody served as loading control. (**D**) Same experiment as in (A), but with a [*PSI*^+^] strain with a C-terminal aid-tag in SEC21 (RK1i). (**E**) Quantification as in (B), but with a [*PSI*^+^] strain with a C-terminal aid-tag in SEC21 (RK1i). Scale bar = 2 μm.
(PDF)

S10 Fig Knock out of proteins required for macroautophagy does not influence the morphology of the IPOD. **(A)** Strains that contained deletions in the endogenous prion domain in SUP35 and express PrD-GFP constitutively under control of the GPD promoter and propagate the prion state and were either wild type (wt) or contained the indicated deletions were used. Cells were grown to logarithmic growth phase, fixed and subjected to fluorescence microscopy. Scale bar = 2μm. **(B)** A strain expressing tGnd1-GFP (# 47 in strain list) was grown to mid log phase and 100 μg/ml of cycloheximide (CHX) was added. At "time 0h", an aliquot for determination of tGnd1-GFP levels by Western Blotting was withdrawn. Subsequently, the culture was split into two aliquots. One was left untreated (- MG132) and MG132 (80 μM) was added to the second (+ MG132). After 8 h of further incubation, aliquots were withdrawn and analyzed by Western Blotting with an antibody against GFP. An anti-actin antibody served as loading control.
(PDF)

S1 Table Proteins identified to bind to PrD (SUP35) fibers. *In vitro* assembled recombinant PrD amyloid fibrils immobilized through a biotin moiety to magnetic avidin beads and incubated them with [*PSI*^+^] yeast cell lysates. Proteins bound to the resin were eluted and subjected to SDS PAGE prior to identification by mass spectrometry (LC-MS/MS). Given are the proteins identified, the size of the corresponding proteins, the number of peptides identified in a control column without immobilized PrD fibrils (control) and the number of peptides identified from a resin with immobilized PrD (+ fibers). Highlighted in green are genes that were known to be related to [*PSI*^*+*^] prion biology; highlighted in yellow are genes that were tested in more detail in this study.
(PDF)

S2 Table Strains used in this study. S2 Table lists all the strains that have been used throughout this study.
(PDF)

S3 Table Plasmids used in this study. S3 Table lists all the plasmids that have been used throughout this study.
(PDF)
