# Supplementary figures and images for: Prion Aggregates Are Recruited to the Insoluble Protein Deposit (IPOD) via Myosin 2-Based Vesicular Transport
Source: PLoS Genet. 2016 Sep 30;12(9):e1006324. doi: 10.1371/journal.pgen.1006324 (PMC5045159; doi:10.1371/journal.pgen.1006324)

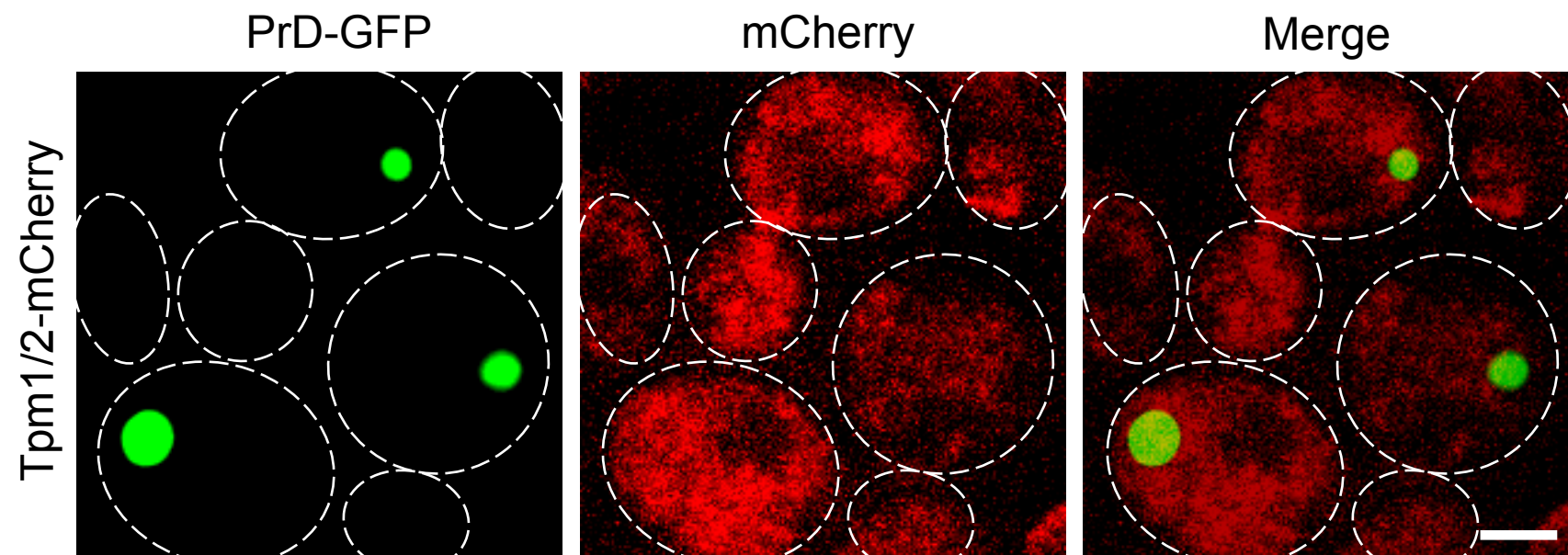

Supplement: S1 Fig — (A) Co-localization of mCherry fusions of tpm1/2 with PrD-GFP in 74D-694-ΔPrD (SUP35) [PrD-GFP+] strain (RK3-TPM1/2-mCh). Logarithmic growth phase culture was fixed and analyzed by fluorescence microscopy as described in methods. After deconvolution, a merged image of the z-stacks taken in the GFP- and the mCherry channels, respectively, were overlaid. Scale bar = 2 μm. (PDF) [file pgen.1006324.s001.pdf]

**A**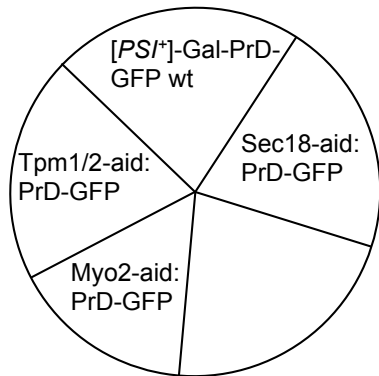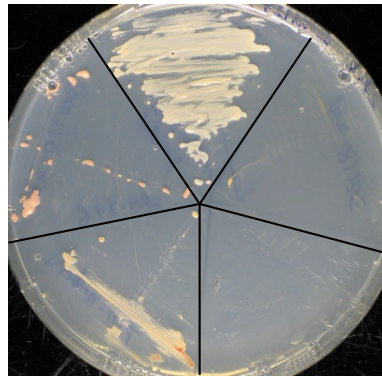

SD + 5mM auxin  
2 days, 30°C

**B**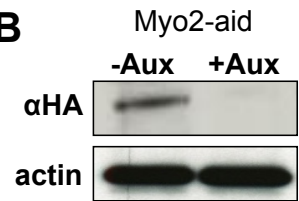**C**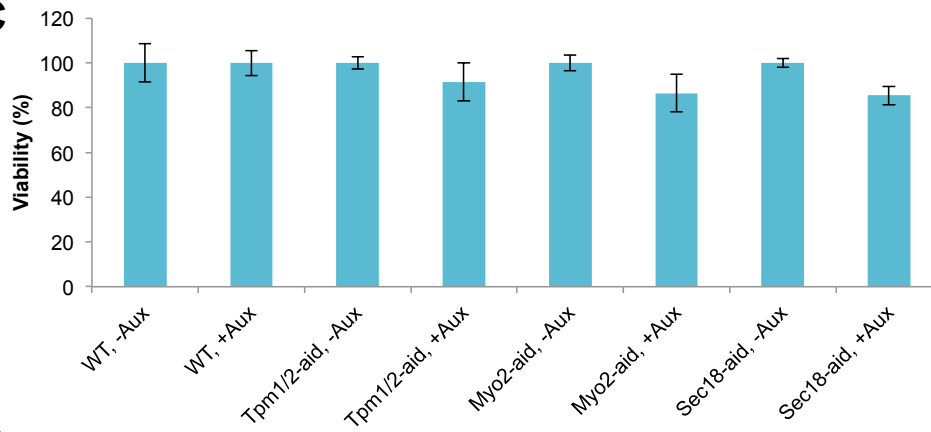**D**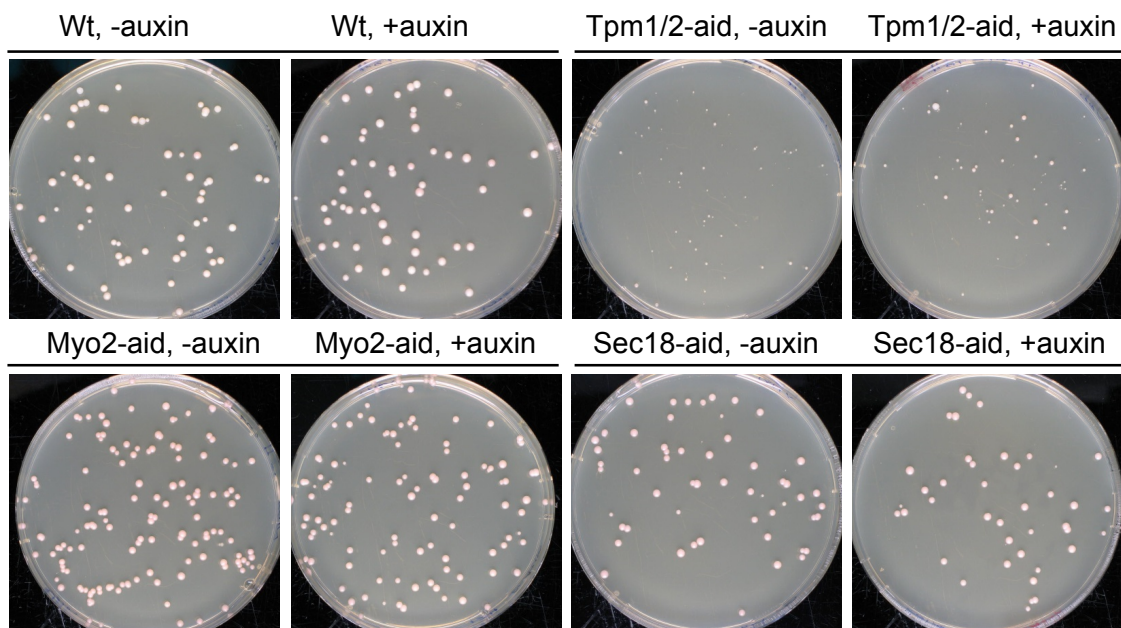

Supplement: S2 Fig — (A) Wild type (wt), tpm1Δ Tpm2-aid, Myo2-aid and Sec18-aid strains (RK1, RK1c, RK1e, RK1g) were streaked onto SD-ura-leu plates containing 5 mM auxin and incubated for 2 days at 30° C. (B) Western blot analysis of a Myo2-aid strain (RK1e) without depletion (- Aux) or after auxin-based depletion (+ Aux) for 6 hours. Myo2 was detected with an antibody against an HA-tag present in the aid-degron-tag. An anti-actin antibody was used as loading control. (C) Quantitative determination of cell viability by Colony-Forming Units (CFU) Assay upon Tpm1/2, Myo2, and Sec18 depletion. Percentage viability of a [PSI+] strains with different C-terminal aid-tag in MYO2, TPM1/2 and SEC18 and the [PSI+] wild type strain (wt). PrD-GFP was induced with galactose for 6 hours in the absence or presence of 20 mM auxin. After 6 hrs of depletion, cultures were serially diluted in YPD from OD600 of 1.0 and 100 μl from the last two dilutions (10−4 and 10−5) were spread on YPD plates and grown at 30°C for 2 days for counting the resulting colonies formed by viable cells. The data are represented as means S.D. of three independent biological replicates. (D) Corresponding pictures of colonies formed by viable cells as in C. (PDF) [file pgen.1006324.s002.pdf]

**A**

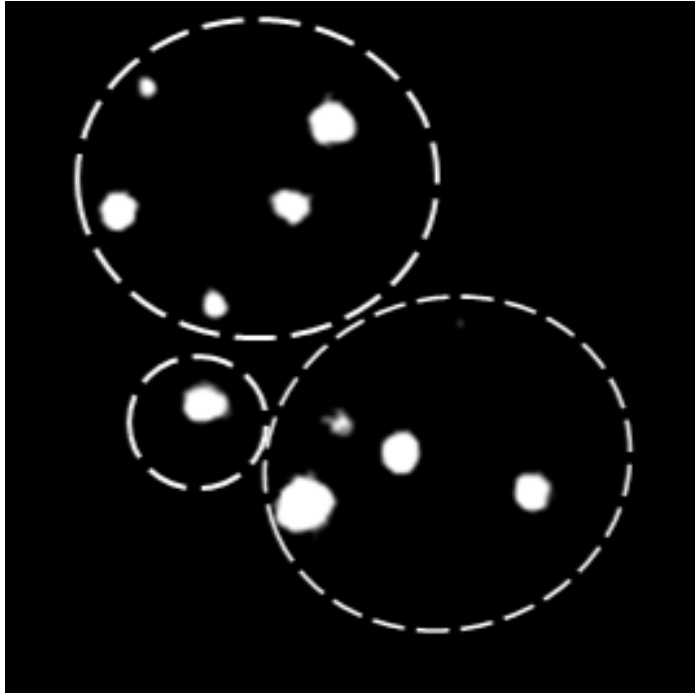

**Captured image of movie S3A**

**B**

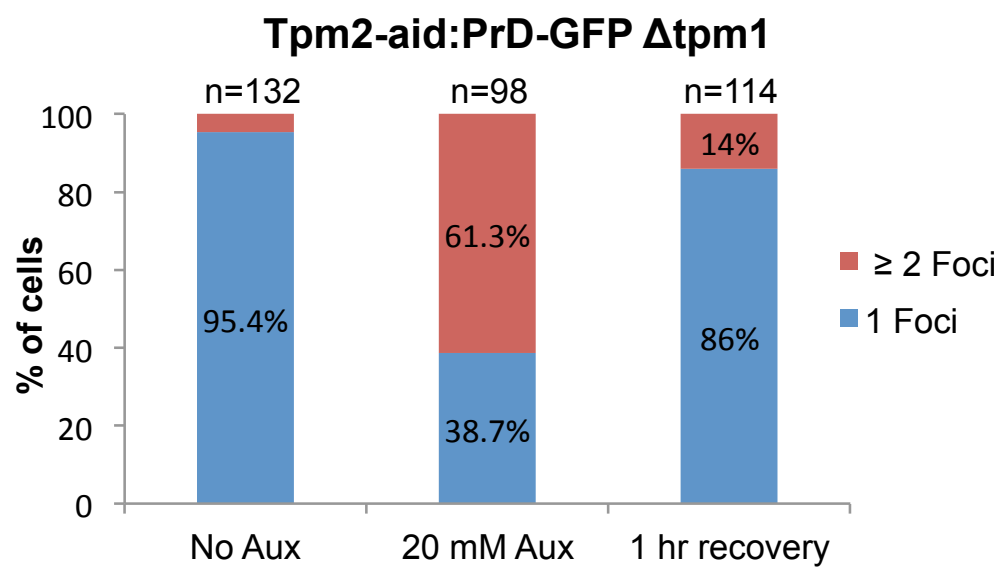

Supplement: S3 Fig — (A) Movie showing refusion of multiple PrD-GFP foci into one single IPOD upon washout of auxin. PrD-GFP was induced with galactose for 6 hours in the presence of 20 mM auxin in a [PSI+] Myo2-aid strain (RK1e). Subsequently, cells were pelleted, resuspended in YPD (glucose chase) and placed onto a microscope slide with a little agarose pad for time-lapse microscopy. Z-stacks with a step width of 0.3 μm were acquired every 2–5 min during one hour. Images were subsequently deblurred using the Wiener Filter algorithm, merged into 1 layer and combined into a movie. (B) PrD-GFP was induced with galactose for 6 hours in a [PSI+] strain containing a deletion of Tpm1 and a C-terminal aid tag in Tpm2 (RK1c). Subsequently, cells were pelleted, resuspended in YPD without auxin to rescue Tpm1/2 function and further incubated for 60 min prior to fixation and microscopic analysis. Subsequently, PrD-GFP localization in either 1 or more than 1 foci was determined and plotted as % in the absence or presence of 20 mM auxin or after auxin washout (1 hr recovery). (PDF) [file pgen.1006324.s003.pdf]

**A****Myo2-aid:PrD-GFP + glucose + auxin**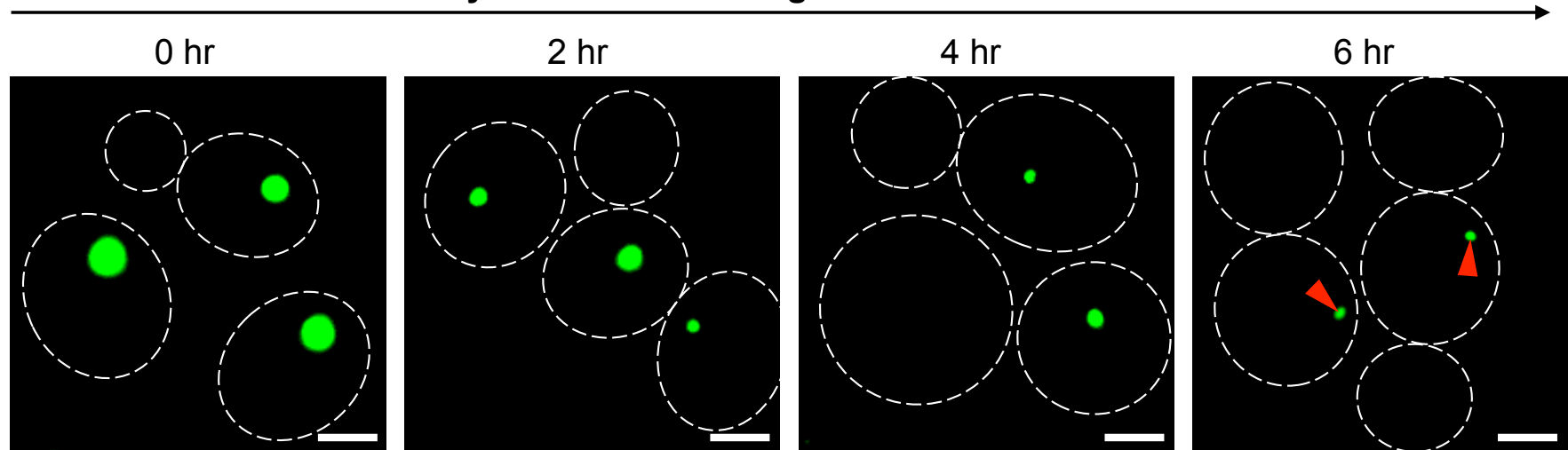**B****Myo2-aid:PrD-GFP + galactose +  
auxin**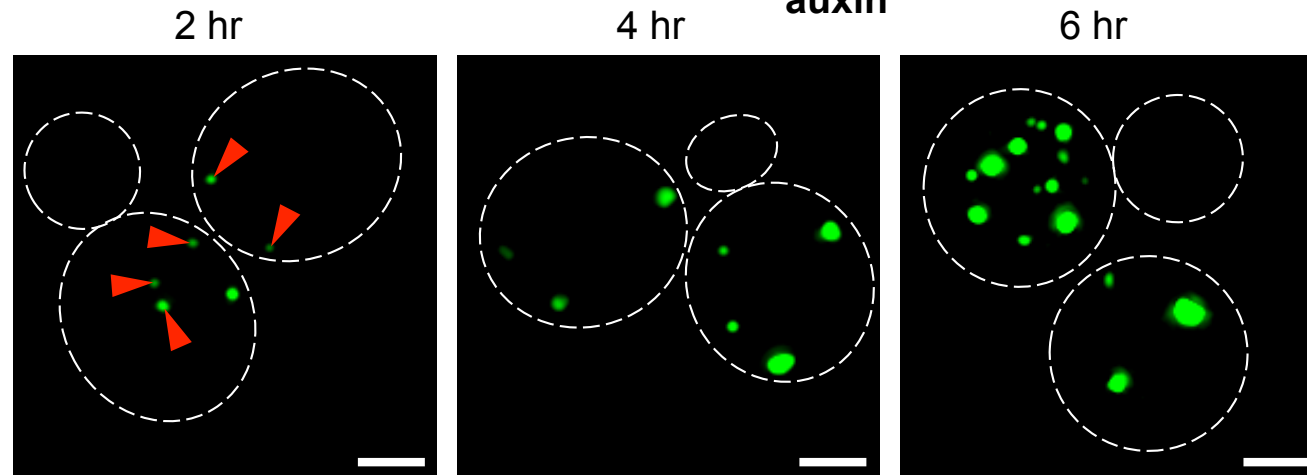

Supplement: S4 Fig — (A) Fluorescence microscopy images for the experiment shown in Fig 3E: PrD-GFP was induced with galactose for 6 hours in the absence of auxin in a [PSI+] Myo2-aid strain (RK1e), cells were pelleted and resuspended in YPD media in the presence of 20 mM auxin. Aliquots were withdrawn every 2 hours, fixed and analyzed by fluorescence microscopy. (B) Fluorescence microscopy images for the experiment shown in Fig 3F: PrD-GFP was induced with galactose in the presence of 20 mM auxin in a [PSI+] Myo2-aid strain (RK1e). After the indicated times, aliquots were withdrawn, fixed and analyzed by fluorescence microscopy. Scale bar = 2μm. (PDF) [file pgen.1006324.s004.pdf]

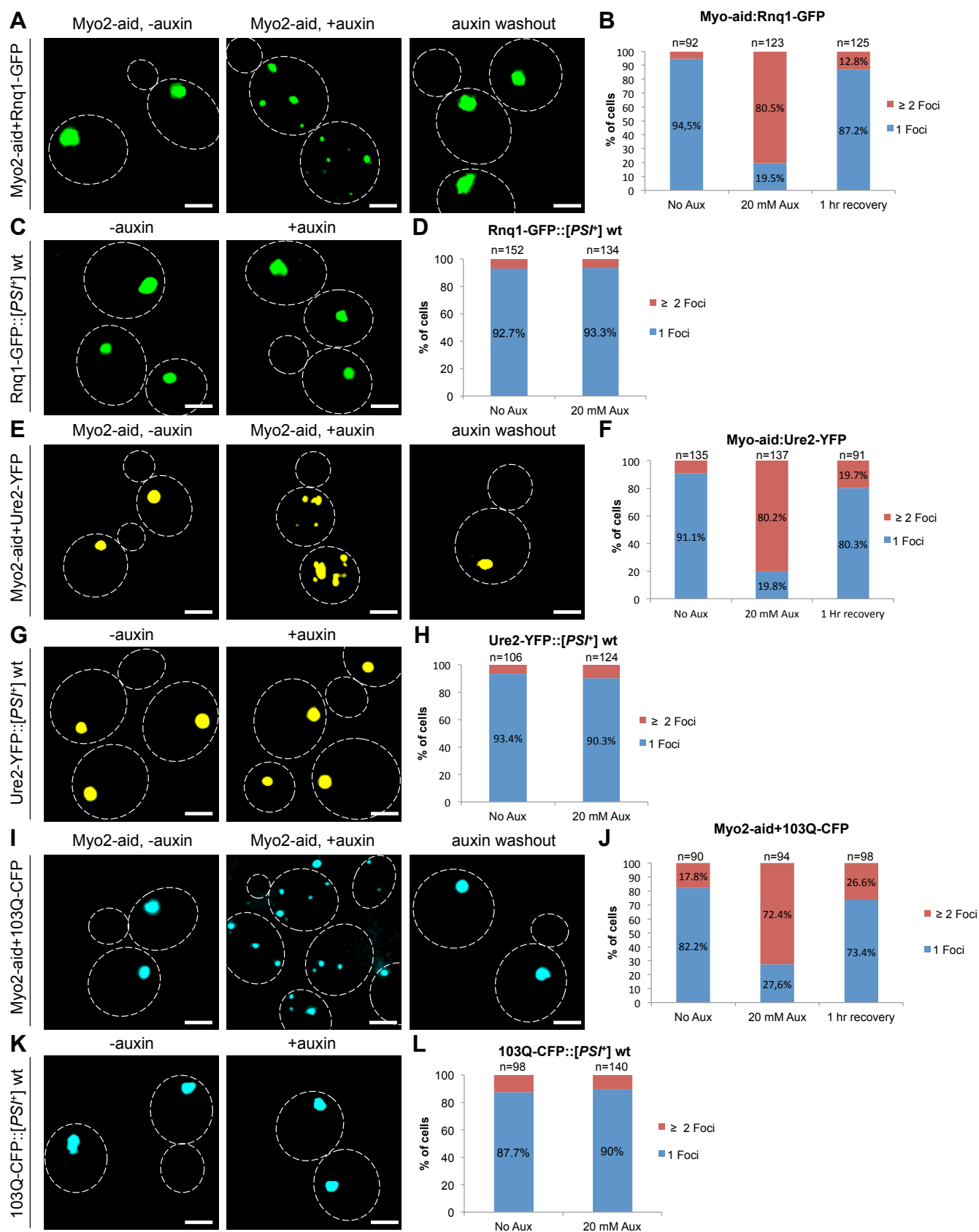

Supplement: S5 Fig — (A, C) Rnq1-GFP was induced with galactose for 6 hours in a [PSI+] strain with a C-terminal aid-tag in MYO2 (RK5b Rnq1-GFP) (left and middle panel) (A) or a wild type (wt) strain (RK6) (C), in the absence or presence of 20 mM of auxin as indicated. Subsequently, cells were pelleted, resuspended in YPD (glucose chase) without auxin to restore Myo2 function and incubated further for 60 min (auxin washout, right panel, (A)) prior to fixation and fluorescence microscopy. (B, D) Quantification of Rnq1-GFP foci upon depletion of Myo2 (B) or in the wild type (wt) (D). Frequencies of cells with 1 single focus or more than 1 foci are given in %. (E, G) Same experiment as in (A), but in a [PSI+] Myo2-aid strain (E) or a wild type strain (G) with a Ure2-YFP construct integrated into the genome under control of the Gal1 promoter (RK5b Ure2-YFP, RK7) (F, H) Quantification as in (B and D), but in a [PSI+] Myo2-aid strain with Ure2-YFP integrated into the genome under control of the Gal1 promoter. (I, K) Same experiment as in (A), but in a [PSI+] Myo2-aid strain or a wild type (wt) strain with a Htt103Q-CFP construct integrated into the genome under control of the Gal1 promoter (RK5b 103Q-CFP, RK8) (J, L) Quantification as in (B and D), but in a [PSI+] Myo2-aid strain with a Htt103Q-CFP construct integrated into the genome under control of the Gal1 promoter. Scale bar = 2 μm. (PDF) [file pgen.1006324.s005.pdf]

**A**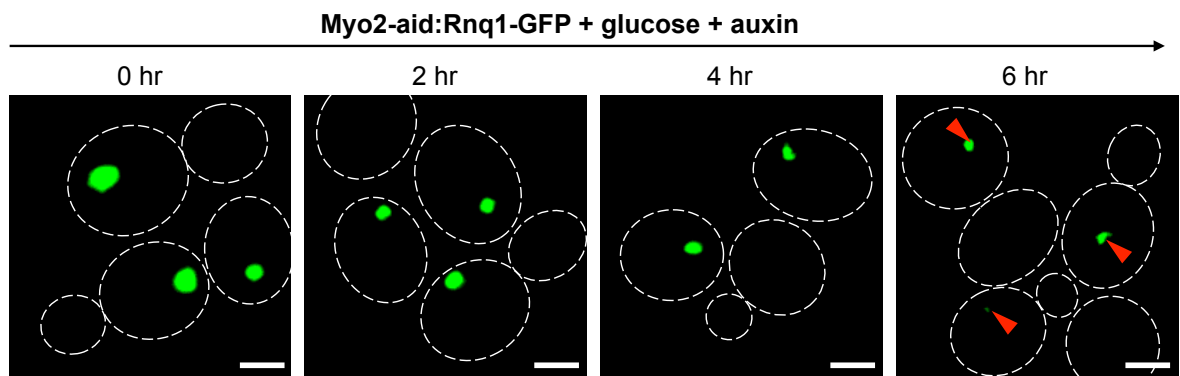**B**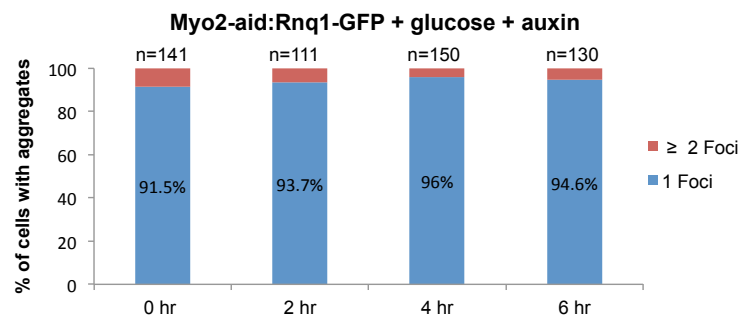**C**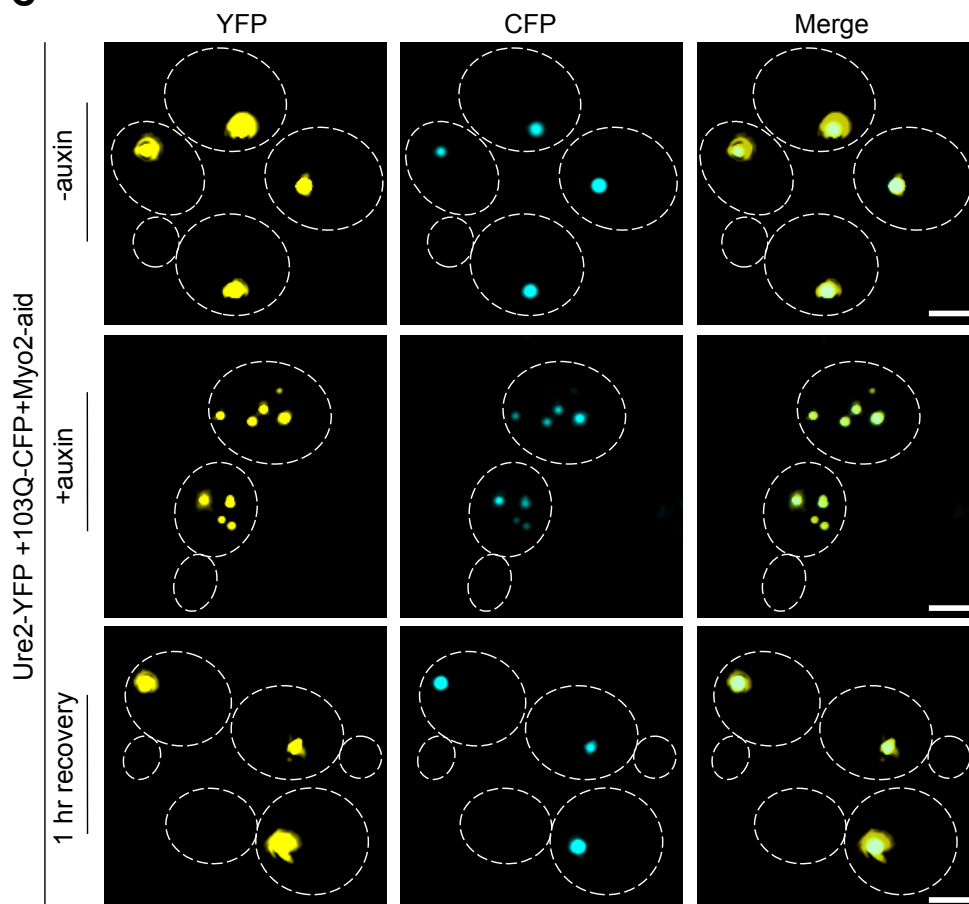

Supplement: S6 Fig — (A) Rnq1-GFP was induced with galactose for 6 hours in the absence of auxin in a [PSI+] Myo2-aid strain (RK5b Rnq1-GFP), cells were pelleted and resuspended in YPD media in the presence of 20 mM auxin. Aliquots were withdrawn every 2 hours, fixed and analyzed by fluorescence microscopy. (B) Quantification of Rnq1-GFP foci that corresponds to S6A Fig. Rnq1-GFP localization in either 1 or more than 1 foci was determined from cells that still carried Rnq1-GFP aggregates and plotted as %. (C) Co-localization of Ure2-YFP with Htt103Q-CFP. Both Ure2-YFP and Htt103Q-CFP were induced with galactose for 6 hours in the absence or presence of 20 mM auxin in a [PSI+] Myo2-aid strain (RK9) (upper and middle panel). Subsequently, cells were pelleted, resuspended in YPD without auxin to restore Myo2 function and incubated further for 60 min (auxin washout, bottom panel) prior to fixation and fluorescence microscopy. Co-localization:–auxin, +auxin and auxin washout: 100%, n = 100–150 foci. Scale bar = 2 μm. (PDF) [file pgen.1006324.s006.pdf]

**A**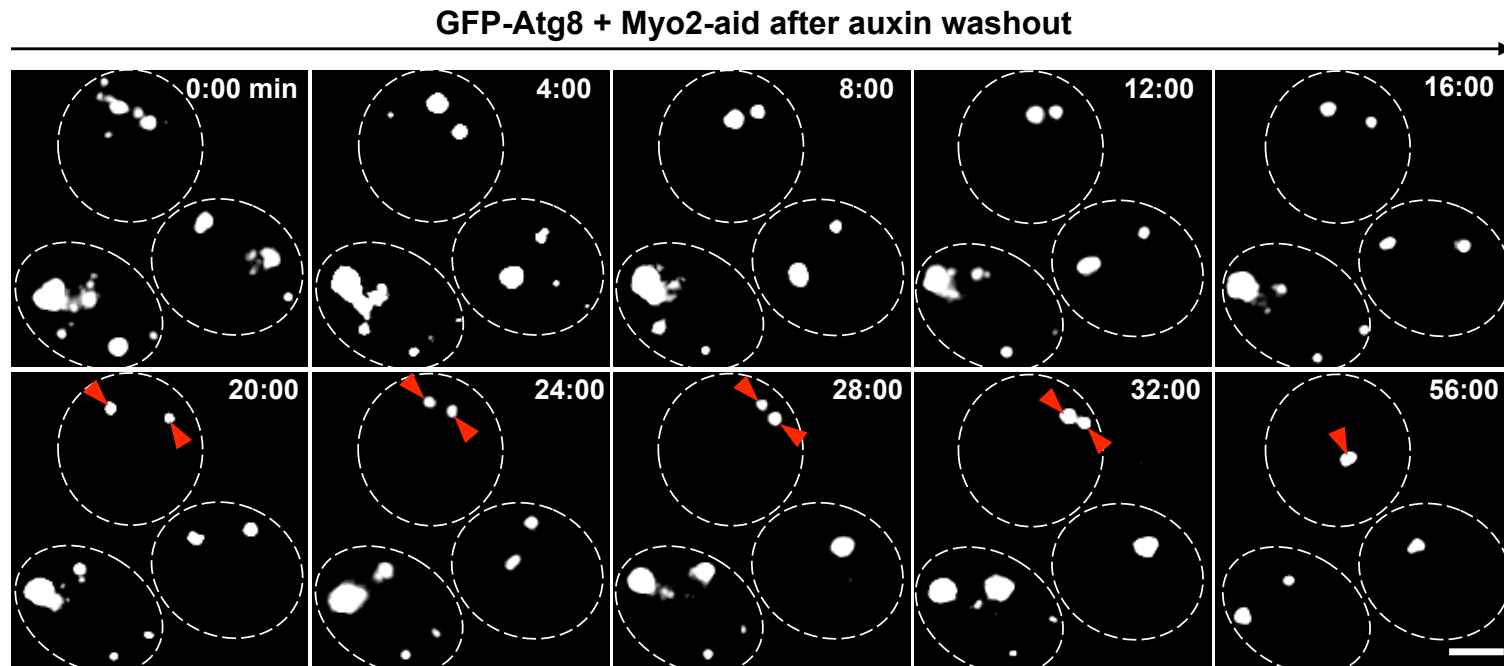**B**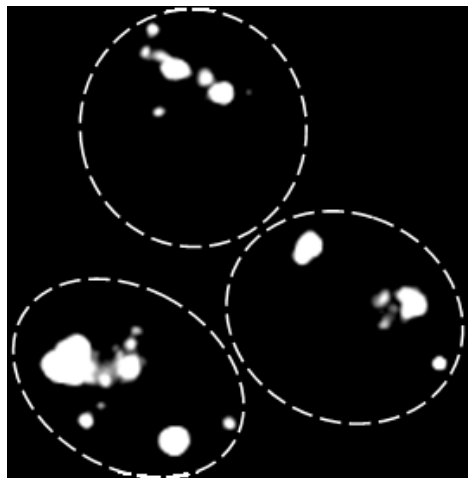

Captured image of movie S7B

**C**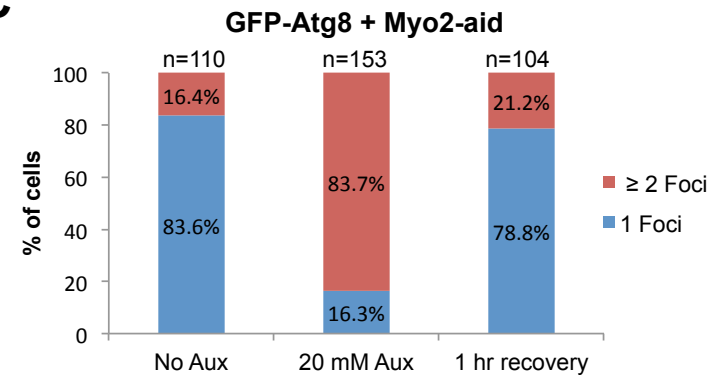

Supplement: S7 Fig — (A) Expression of GFP-Atg8 was induced with galactose for 6 hours in the presence of 20 mM auxin in a [PSI+] Myo2-aid strain containing an N-terminal genomic GFP-fusion to Atg8 under control of the Gal promoter (RK5f). Cells were pelleted, resuspended in YPD in the absence of auxin to restore Myo2 function and placed onto a microscope slide with an agarose pad for time-lapse microscopy. Z-stacks with a step width of 0.2 μm were acquired every 4 min. Images were deblurred using the Wiener Filter algorithm and combined into one image. (B) Movie showing re-fusion of GFP-Atg8 foci generated during Myo2 depletion and subsequent washout of auxin as described in (A). (C) Quantification of GFP-Atg8 localization in either 1 or more than 1 foci in % in the absence or presence of 20 mM auxin or after auxin washout (1 hr recovery). Scale bar = 2μm. (PDF) [file pgen.1006324.s007.pdf]

[PSI<sup>+</sup>]-Gal-PrD-GFP wt

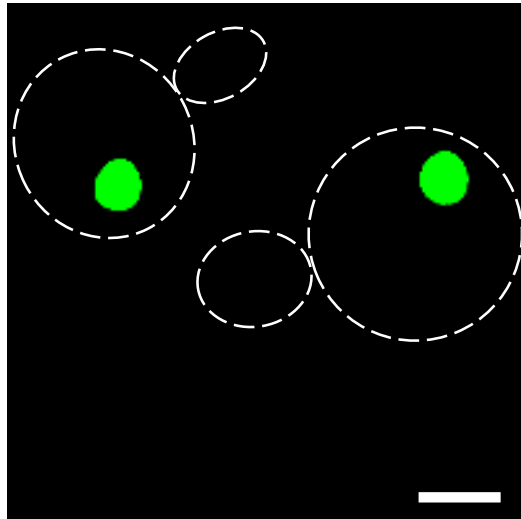

$\Delta$ ape1

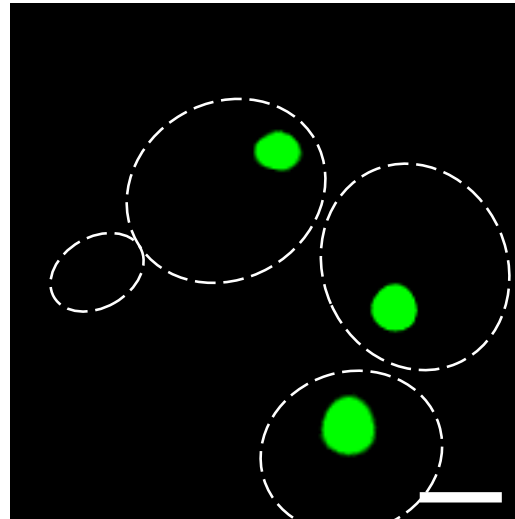

$\Delta$ ams1

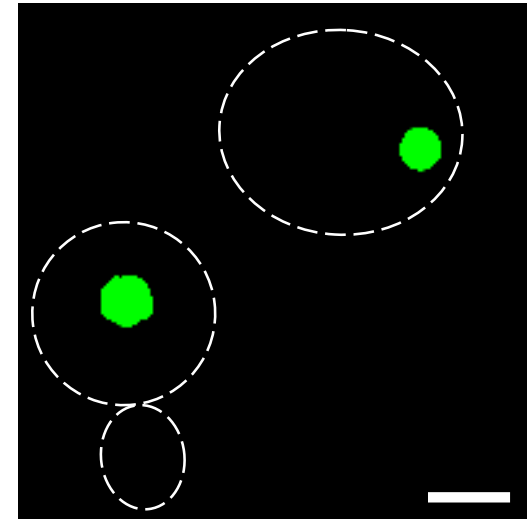

$\Delta$ atg9

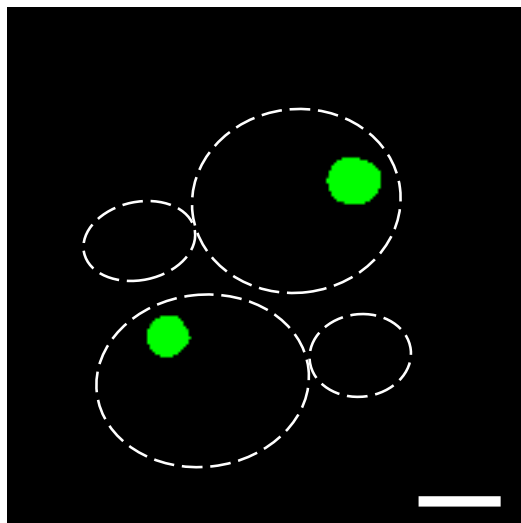

$\Delta$ atg11

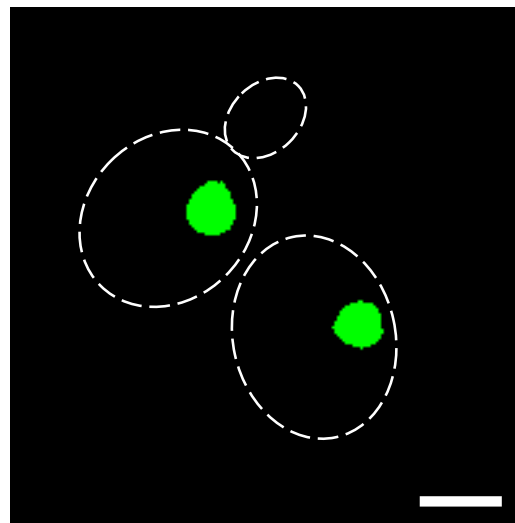

$\Delta$ atg19

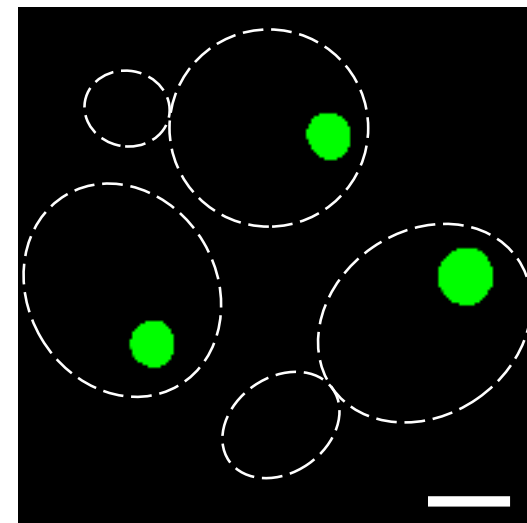

Supplement: S8 Fig — PrD-GFP was induced with galactose for 6 hours in a [PSI+] wt strain or an identical strain, but with the indicated deletions. Cells were fixed and analyzed by fluorescence microscopy. Scale bar = 2μm. (PDF) [file pgen.1006324.s008.pdf]

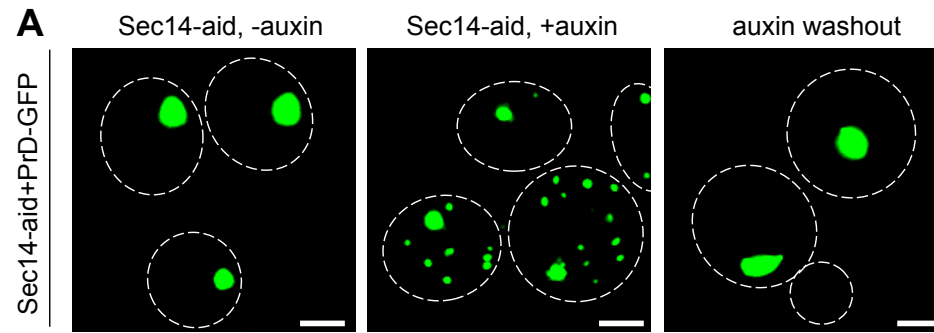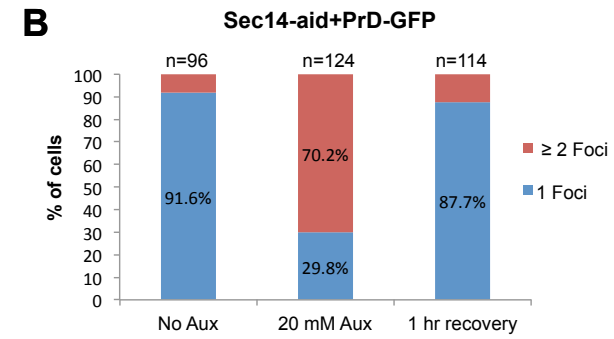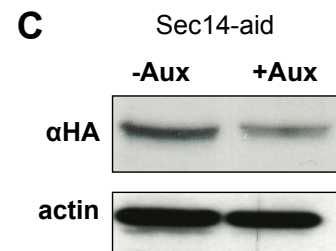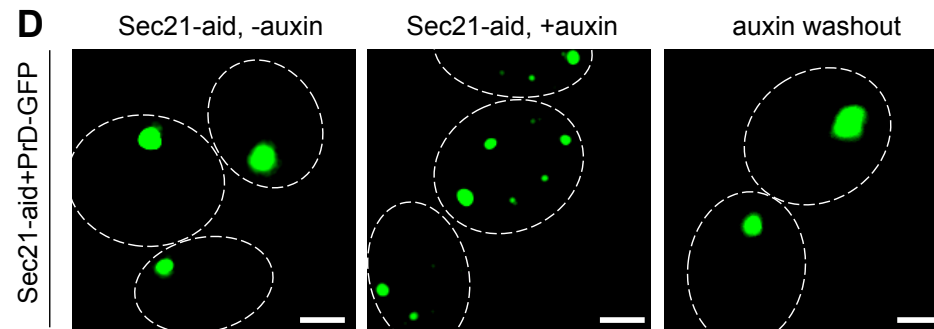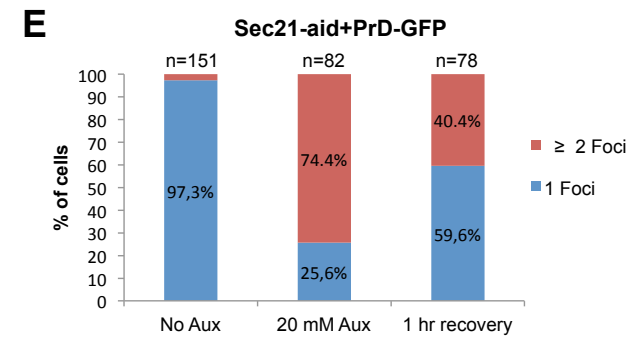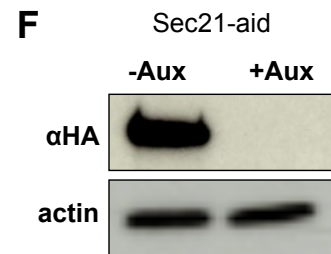

Supplement: S9 Fig — (A) PrD-GFP was induced with galactose for 6 hours in the absence or presence of 20 mM auxin (left and middle panel) in a [PSI+] strain with a C-terminal aid-tag in SEC14 (RK1h). Subsequently, cells were pelleted, resuspended in YPD (glucose chase) without auxin to restore Sec14 function and incubated further for 60 min (auxin washout, right panel) prior to fixation and fluorescence microscopy. (B) Quantification of PrD-GFP foci upon depletion of Sec14. Frequencies of cells with 1 single focus or more than 1 foci are given in %. Scale bar = 2 μm. (C, F) Western blot analysis of a [PSI+] Sec14-aid and Sec21-aid strain without (-Aux) or after depletion (+Aux) of Sec14 and Sec21 with an antibody against an HA-tag present in the aid-tag. An anti-actin antibody served as loading control. (D) Same experiment as in (A), but with a [PSI+] strain with a C-terminal aid-tag in SEC21 (RK1i). (E) Quantification as in (B), but with a [PSI+] strain with a C-terminal aid-tag in SEC21 (RK1i). Scale bar = 2 μm. (PDF) [file pgen.1006324.s009.pdf]

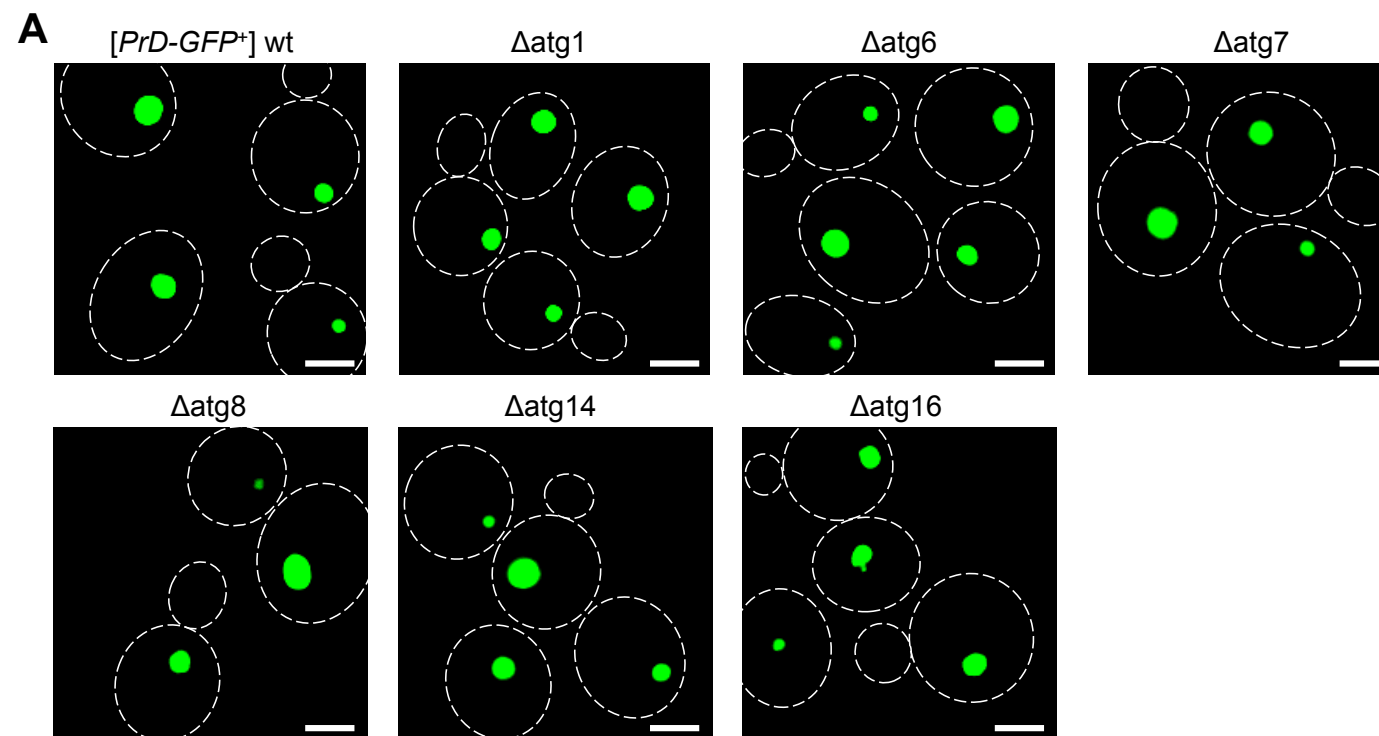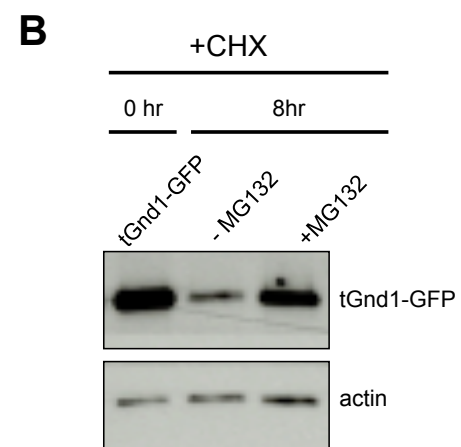

Supplement: S10 Fig — (A) Strains that contained deletions in the endogenous prion domain in SUP35 and express PrD-GFP constitutively under control of the GPD promoter and propagate the prion state and were either wild type (wt) or contained the indicated deletions were used. Cells were grown to logarithmic growth phase, fixed and subjected to fluorescence microscopy. Scale bar = 2μm. (B) A strain expressing tGnd1-GFP (# 47 in strain list) was grown to mid log phase and 100 μg/ml of cycloheximide (CHX) was added. At "time 0h", an aliquot for determination of tGnd1-GFP levels by Western Blotting was withdrawn. Subsequently, the culture was split into two aliquots. One was left untreated (- MG132) and MG132 (80 μM) was added to the second (+ MG132). After 8 h of further incubation, aliquots were withdrawn and analyzed by Western Blotting with an antibody against GFP. An anti-actin antibody served as loading control. (PDF) [file pgen.1006324.s010.pdf]
