# Supplementary material for: Prion Aggregates Are Recruited to the Insoluble Protein Deposit (IPOD) via Myosin 2-Based Vesicular Transport
Source: PLoS Genet. 2016 Sep 30;12(9):e1006324. doi: 10.1371/journal.pgen.1006324 (PMC5045159; doi:10.1371/journal.pgen.1006324)
Supplement: S1 Table — In vitro assembled recombinant PrD amyloid fibrils immobilized through a biotin moiety to magnetic avidin beads and incubated them with [PSI+] yeast cell lysates. Proteins bound to the resin were eluted and subjected to SDS PAGE prior to identification by mass spectrometry (LC-MS/MS). Given are the proteins identified, the size of the corresponding proteins, the number of peptides identified in a control column without immobilized PrD fibrils (control) and the number of peptides identified from a resin with immobilized PrD (+ fibers). Highlighted in green are genes that were known to be related to [PSI+] prion biology; highlighted in yellow are genes that were tested in more detail in this study. (PDF) [file pgen.1006324.s011.pdf]

S1 Table: Proteins identified to bind to PrD (SUP35) fibers

Identified protein	Size (kDa)	Number of peptides found	
		control	+ fibers
ULP1	72	2	11
PAB1	64	1	28
PUB1	51	-	8
NAP1	48	1	6
CHA1	39	-	2
SIS1	38	-	3
RPL2A	27	-	4
RPS3	27	3	13
SMT3	12	2	5
SSA2	69	12	13
HRP1	60	0	6
VMA2	58	3	8
DHH1	58	1	3
ATP2	55	3	6
TUB2	51	10	14
YDJ1	45	-	6
NSR1	45	1	6
LYS12	40	8	16
PSA1	40	5	10
ADH1	37	10	12
SGT2	37	15	18
IDH2	40	4	9
AAH1	40	6	8
SUA7	38	2	5
PRS3	34	11	18
RPS4a	29	4	9
VMA8	29	1	5
RPS6A	27	1	4
YKT6	23	6	15
RPS17A	17	3	5
HYP2	17	2	4
DNM1	85	4	9
SEC18	84	3	7
SSA1	70	-	1
PDR16	41	1	10
FUS3	41	-	4
IDH1	39	3	10
GET3	39	3	9
ASC1	35	2	10
KTI12	35	4	7
RPL5	34	1	5
PRO3	30	2	7
RVS161	30	3	4
SNO1	25	1	5
GSP2	25	0	1
TPM1	24	-	5
YPT1	23	1	2
SAR1	21	7	8
APT2	20	2	4

SLA2	109	0	3
LSB3	49	1	4
SEC14	35	1	5
GCS1	39	-	6
NPL3	45	-	7
SEC4	24	5	7
SEC21	105	2	3
SEC53	29	1	5
NRD1	64	-	9
RPL6B	20	0	5
RHB1	23	1	5
RPL20A	20	1	5
TPM2	19	-	2
NEW1	134	2	4
RPS1A	29	3	6

Related to [*PSI*$^+$] prion biology

Tested in more detail in this study
