# Supplementary material for: Prion Aggregates Are Recruited to the Insoluble Protein Deposit (IPOD) via Myosin 2-Based Vesicular Transport
Source: PLoS Genet. 2016 Sep 30;12(9):e1006324. doi: 10.1371/journal.pgen.1006324 (PMC5045159; doi:10.1371/journal.pgen.1006324)
Supplement: S2 Table — lists all the strains that have been used throughout this study. (PDF) [file pgen.1006324.s012.pdf]

**S2 Table: Strains used in this study**

| S.N. | NAME | GENOTYPE | SOURCE/REFERENCE |
|---|---|---|---|
| 1 | RK1 | 74D-694-[*PSI*$^+$]-Gal-PrD-GFP::LEU2 | (1) |
| 2 | RK1a | RK1 tpm1Δ::natNT2 | This study |
| 3 | RK1b | RK1a-OsTIR1::URA3 | This study |
| 4 | RK1c | RK1b-TPM2-AID ::*kanMX4* | This study |
| 5 | RK1d | RK1-OsTIR1::URA3 | This study |
| 6 | RK1e | RK1d-MYO2-AID::kanMX4 | This study |
| 7 | RK1g | RK1d-SEC18-AID::kanMX4 | This study |
| 8 | RK1 atg9Δ | RK1 atg9::hphNT1 | This study |
| 9 | RK1 atg11Δ | RK1 atg11::natNT2 | This study |
| 10 | RK1 atg19Δ | RK1 atg19:natNT2 | This study |
| 11 | RK1 ape1Δ | RK1 ape1::kanMX4 | This study |
| 12 | RK1 ams1Δ | RK1 ams1::kanMX4 | This study |
| 13 | RK1 sgt2Δ | RK1 sgt2::kanMX4 | This study |
| 14 | RK1e + pRFP-ATG8 | RK1e + pRS414 RFP-ATG8 | This study |
| 15 | RK1e APE1-mCh | RK1e-303 APE1-mCh::HIS3 | This study |
| 16 | RK1e ATG9-3xmCh | RK1e-ATG9-3xmCh::hph NT1 | This study |
| 17 | RK1g + pmCh-ATG8 | RK1g + pRS413 mCh-ATG8 | This study |
| 18 | RK1g MYO2-3xmCh | RK1g-MYO2-3xmCh::hph NT1 | This study |
| 19 | RK3 | 74D-694-ΔPrD (SUP35) [*PrD-GFP*$^+$]::TRP1 | (1) |
| 20 | RK3 TPM1-mCh | RK3-TPM1-mCh::hphNT1 | This study |
| 21 | RK3 TPM2-mCh | RK3-TPM2-mCh::hphNT1 | This study |
| 22 | RK3 atg1Δ | RK3 atg1Δ::natNT2 | This study |
| 23 | RK3 atg6Δ | RK3 atg6Δ::natNT2 | This study |
| 24 | RK3 atg7Δ | RK3 atg7Δ::natNT2 | This study |
| 25 | RK3 | RK3 atg8Δ::natNT2 | This study |

| | | | |
|---|---|---|---|
| | atg8Δ | | |
| 26 | RK3 atg14Δ | RK3 atg14Δ::natNT2 | This study |
| 27 | RK3 atg16Δ | RK3 atg16Δ::natNT2 | This study |
| 28 | RK3 pep4Δ | RK3 pep4Δ::natNT2 | This study |
| 29 | RK4 | 74D-694-ΔPrD (SUP35) [*prd-gfp*$^{-}$]::TRP1 | (1) |
| 30 | RK4 pep4Δ | RK4 pep4Δ::natNT2 | This study |
| 31 | 74D | 74D-694-[*PSI*$^{+}$] | (2) |
| 32 | RK5a | 74D-OsTIR1::URA3 | This study |
| 33 | RK5b | RK5a-MYO2-AID::kanMX4 | This study |
| 36 | RK5e | RK5b-GFP-preApe1::natNT2 | This study |
| 37 | RK5f | RK5b-GFP-ATG8::natNT2 | This study |
| 38 | RK5b Rnq1-GFP | RK5b-305 Gal RNQ1-GFP::LEU2 | This study |
| 39 | RK5b Ure2-YFP | RK5b-305 Gal URE2-YFP::LEU2 | This study |
| 40 | RK5b 103Q-CFP | RK5b-304 Gal 103Q-CFP::TRP1 | This study |
| 41 | RK6 | 74D-305 Gal RNQ1-GFP::LEU2 | This study |
| 42 | RK7 | 74D-305 Gal URE2-YFP::LEU2 | This study |
| 43 | RK8 | 74D-304 Gal 103Q-CFP::TRP1 | This study |
| 44 | RK1h | RK1d-SEC14-AID::kanMX4 | This study |
| 45 | RK1i | RK1d-SEC21-AID::kanMX4 | This study |
| 46 | RK1j | RK1d-SEC53-AID::kanMX4 | This study |
| 47 | tGnd1-GFP | BY4741-tGnd1-GFP:: natNT2 Δprd5 ::kanMX4 | (3) |
| 48 | RK1 Δprd5 | 74D-694-[*PSI*$^{+}$]-Gal-PrD-GFP::LEU2 Δprd5::hph NT1 | This study |
| 49 | RK9 | RK5b-304 Gal 103Q-CFP::TRP1-305 Gal URE2-YFP::LEU2 | This study |

1. Tyedmers J, Treusch S, Dong J, McCaffery JM, Bevis B, Lindquist S. Prion induction involves an ancient system for the sequestration of aggregated proteins and heritable changes in prion fragmentation. Proceedings of the National Academy of Sciences of the United States of America. 2010 May 11;107(19):8633-8. PubMed PMID: 20421488. Pubmed Central PMCID: 2889312.
2. Chernoff YO, Lindquist SL, Ono B, Inge-Vechtomov SG, Liebman SW. Role of the chaperone protein Hsp104 in propagation of the yeast prion-like factor [psi+]. Science. 1995 May 12;268(5212):880-4. PubMed PMID: 7754373. Epub 1995/05/12. eng.
3. Miller SB, Ho CT, Winkler J, Khokhrina M, Neuner A, Mohamed MY, et al. Compartment-specific aggregases direct distinct nuclear and cytoplasmic aggregate deposition. The EMBO journal. 2015 Mar 12;34(6):778-97. PubMed PMID: 25672362. Pubmed Central PMCID: 4369314.
