# Supplementary material for: Prion Aggregates Are Recruited to the Insoluble Protein Deposit (IPOD) via Myosin 2-Based Vesicular Transport
Source: PLoS Genet. 2016 Sep 30;12(9):e1006324. doi: 10.1371/journal.pgen.1006324 (PMC5045159; doi:10.1371/journal.pgen.1006324)
Supplement: S3 Table — lists all the plasmids that have been used throughout this study. (PDF) [file pgen.1006324.s013.pdf]

S3 Table: plasmids used in this study

S.N.	NAME	SOURCE/REFERENCE
1	pH10sumo-PrD-TEV-Avi	This study
2	pHis-Sumo-PrD-STOP	This study
3	pRS413-mCherry-Atg8	Nava Segev lab
4	pRS414-RFP-Atg8	Daniel Klionsky lab
5	pRS303-Ape1-mCherry	Yoshinori Ohsumi lab
6	pRS416-GFP-Atg8	Daniel Klionsky lab
7	pBS35	The Yeast Resource Center
8	pFA6a-kanMX4	(1)
9	pFA6a-hphNT1	Michael Knop lab (2)
10	pFA6a-natNT2	Michael Knop lab (2)
11	pYM-N17	Michael Knop lab (2)
12	pNHK53	(3)
13	pMK43	(3)
14	pMaM144 (3 x mCherry)	Micheal Knop lab
15	pRS305 Gal RNQ1-GFP::LEU2	Bernd Bukau lab
16	pRS305 Gal URE2-YFP::LEU2	Bernd Bukau lab
17	pRS304 Gal 103Q-CFP::TRP1	Bernd Bukau lab

1. Wach A, Brachat A, Pohlmann R, Philippsen P. New heterologous modules for classical or PCR-based gene disruptions in Saccharomyces cerevisiae. Yeast. 1994;10(13):1793-808. Epub 1994/12/01.
2. Janke C, Magiera MM, Rathfelder N, Taxis C, Reber S, Maekawa H, et al. A versatile toolbox for PCR-based tagging of yeast genes: new fluorescent proteins, more markers and promoter substitution cassettes. Yeast. 2004;21(11):947-62. Epub 2004/08/31.
3. Nishimura K, Fukagawa T, Takisawa H, Kakimoto T, Kanemaki M. An auxin-based degron system for the rapid depletion of proteins in nonplant cells. Nature methods. 2009;6(12):917-22. Epub 2009/11/17.
